# Reactive astrocytes secrete the chaperone HSPB1 to mediate neuroprotection

**DOI:** 10.1126/sciadv.adk9884

**Published:** 2024-03-20

**Authors:** Fangjia Yang, Paula Beltran-Lobo, Katherine Sung, Caoimhe Goldrick, Cara L. Croft, Agnes Nishimura, Erin Hedges, Farah Mahiddine, Claire Troakes, Todd E. Golde, Beatriz G. Perez-Nievas, Diane P. Hanger, Wendy Noble, Maria Jimenez-Sanchez

**Affiliations:** ^1^ Department of Basic and Clinical Neuroscience, Maurice Wohl Clinical Neuroscience Institute, Institute of Psychiatry, Psychology and Neuroscience, King's College London, 5 Cutcombe Road, London SE5 9RX, UK.; ^2^ UK Dementia Research Institute, UCL Institute of Neurology, University College London, London, UK.; ^3^ Centre for Neuroscience, Surgery and Trauma, Blizard Institute, Barts and the London School of Medicine and Dentistry, Queen Mary University of London, London, UK.; ^4^ London Neurodegenerative Diseases Brain Bank, Institute of Psychiatry, Psychology and Neuroscience, King's College London, London, UK.; ^5^ Department of Neuroscience, College of Medicine, University of Florida, Gainesville, FL, USA.; ^6^ Center for Translational Research in Neurodegenerative Disease, College of Medicine, University of Florida, Gainesville, FL, USA.; ^7^ McKnight Brain Institute, College of Medicine, University of Florida, Gainesville, FL, USA.; ^8^ Department of Pharmacology and Chemical Biology, Department of Neurology, Emory Center for Neurodegenerative Disease, Emory University, Atlanta, GA, USA.; ^9^ Department of Biomedical and Clinical Sciences, University of Exeter, Exeter, UK.

## Abstract

Molecular chaperones are protective in neurodegenerative diseases by preventing protein misfolding and aggregation, such as extracellular amyloid plaques and intracellular tau neurofibrillary tangles in Alzheimer’s disease (AD). In addition, AD is characterized by an increase in astrocyte reactivity. The chaperone HSPB1 has been proposed as a marker for reactive astrocytes; however, its astrocytic functions in neurodegeneration remain to be elucidated. Here, we identify that HSPB1 is secreted from astrocytes to exert non–cell-autonomous protective functions. We show that in human AD brain, HSPB1 levels increase in astrocytes that cluster around amyloid plaques, as well as in the adjacent extracellular space. Moreover, in conditions that mimic an inflammatory reactive response, astrocytes increase HSPB1 secretion. Concomitantly, astrocytes and neurons can uptake astrocyte-secreted HSPB1, which is accompanied by an attenuation of the inflammatory response in reactive astrocytes and reduced pathological tau inclusions. Our findings highlight a protective mechanism in disease conditions that encompasses the secretion of a chaperone typically regarded as intracellular.

## Teaser

The chaperone HSPB1 is a mediator of astrocyte-neuron communication with protective functions in neurodegeneration.

## INTRODUCTION

Astrocyte reactivity is a prominent feature in neurodegeneration, including in Alzheimer’s disease (AD), with astrocytes clustering around dense-core amyloid plaques and with the number of reactive astrocytes increasing as disease advances (*1*). Among the functional, morphological, and transcriptional changes that characterize reactive astrocytes (*2*), an increase in the levels of the small heat shock protein (sHSP) HSPB1 is shared across brain injury and disease conditions (*3*, *4*), and HSPB1 has been proposed as a marker for reactive astrocytes (*2*).

HSPB1 [also referred to as HSP27 (human) or HSP25 (mouse)] is an adenosine triphosphate (ATP)–independent chaperone with an α-crystallin domain flanked by variable N- and C-terminal sequences, a structure that is conserved across the sHSP family (*5*). In human aged and AD brain, transcription of most chaperones is generally repressed, but this is not the case for chaperones belonging to the family of sHSPs, including HSPB1 (*6*). HSPB1 is regarded as protective in neurodegeneration by keeping aggregate-prone proteins in a folding-competent state (*7*–*10*) and by participating in phase separation (*11*, *12*), as well as by exerting other functions that include antiapoptotic (*13*, *14*) and antioxidant properties (*15*), modulation of the cytoskeleton architecture (*16*), and maintenance of mitochondrial proteostasis (*17*). In addition, both pro- and anti-inflammatory roles have been attributed to HSPB1 (*18*). Moreover, when ubiquitously expressed, HSPB1 ameliorated some of the pathological, synaptic, and cognitive symptoms observed in a mouse model of amyloid pathology (*19*). However, the functional implication of HSPB1 in reactive astrocytes remains elusive.

While traditionally considered intracellular proteins, chaperones are also found extracellularly (*20*), and transcellular chaperone signaling is necessary for the maintenance of organismal proteostasis (*21*). A non–cell-autonomous role of chaperones is likely to be very important in neurodegeneration, where a failure of proteostasis in neurons is a prominent feature (*22*). This supports a role for chaperones as mediators of intercellular communication that might be released from astrocytes and taken up by neurons and adjacent cells to strengthen their proteostasis systems.

An effective astrocyte-neuron communication is key to maintenance of a healthy brain, and a better understanding of these actions is essential to gain insight into protective mechanisms in neurodegeneration. Here, we set out to investigate the functional role of HSPB1 in astrocyte-neuron interactions in AD. Our data show that HSPB1 is expressed primarily in astrocytes and accumulates in glial fibrillary acidic protein (GFAP)–positive astrocytes that cluster around amyloid plaques in postmortem AD brain. In particular, we observe an increase in HSPB1 in the extracellular space of astrocytes surrounding Aβ deposits. These data are mirrored in vitro, where astrocytic secretion of HSPB1 is increased in cultured rodent astrocytes subjected to a proinflammatory stimulus. We observe that HSPB1 is released as a membrane-free protein that can be internalized by surrounding cells. By virally expressing human HSPB1 or with recombinant human HSPB1 (rhHSPB1) treatment, we show that extracellular human HSPB1 is protective in mouse organotypic brain slices in response to proinflammatory factors and prevents the accumulation of inclusions in a P301L/S320F mutant tau model of neurofibrillary pathology. This work identifies a unique mechanism by which HSPB1 is secreted from astrocytes in reactive conditions to mediate autocrine and paracrine neuroprotective functions and highlights the therapeutic potential of HSPB1 to ameliorate tau pathology in AD and related tauopathies.

## RESULTS

### HSPB1 expression is increased in amyloid plaque–associated astrocytes and in their surrounding space

**Fig. 1.**
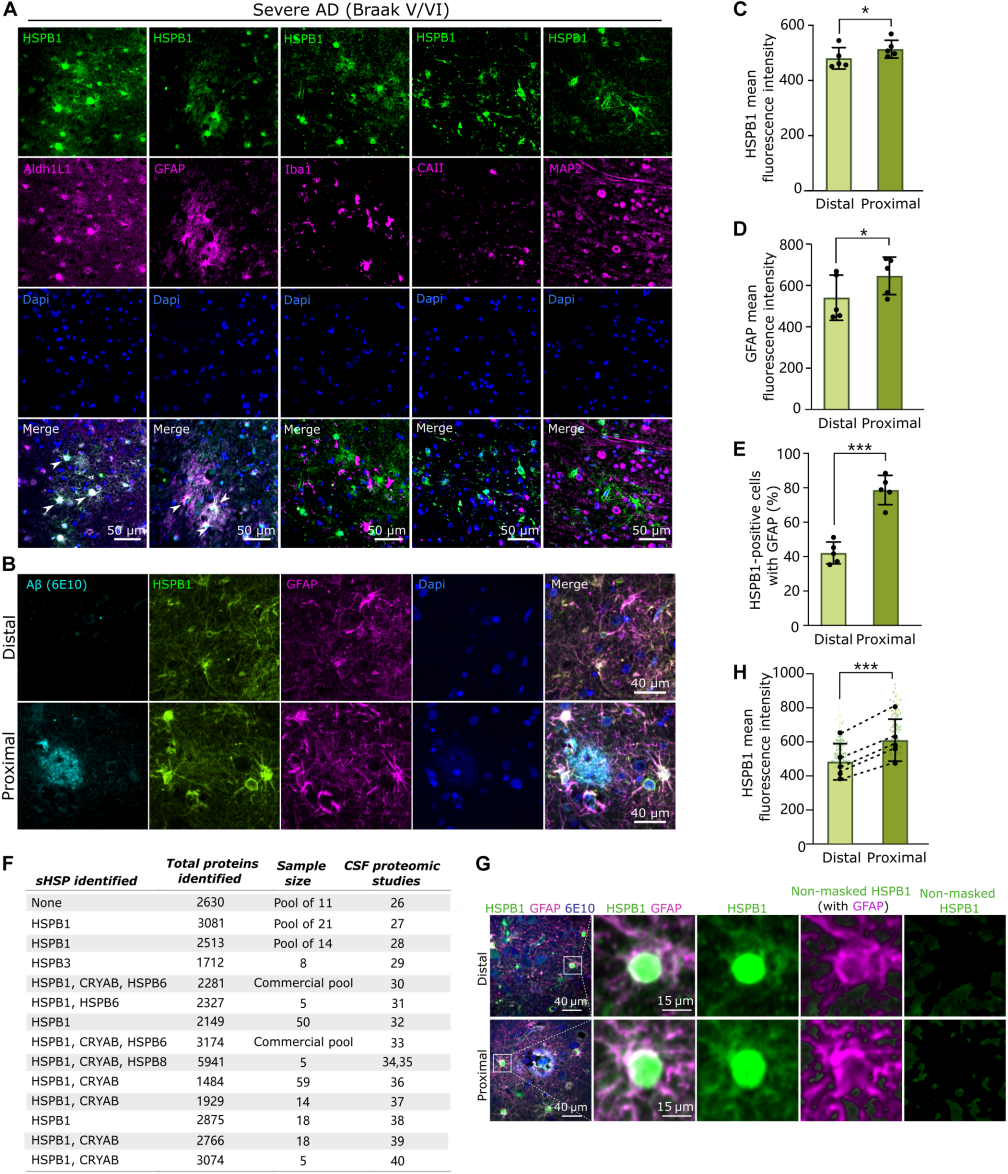
HSPB1 is found in astrocytes proximal to amyloid plaques and in their surrounding space. (**A**) Representative images of temporal cortex from severe cases of AD (Braak V/VI) coimmunostained with HSPB1 and ALDHL1 (astrocytes), GFAP (reactive astrocytes), IBA1 (microglia), CAII (oligodendrocytes), and MAP2 (neurons). (**B**) Representative images of GFAP^+^ and HSPB1^+^ astrocytes clustering in the areas proximal to amyloid plaques (positive for 6E10 anti-Aβ). (**C** and **D**) Graphs show the mean fluorescence intensity of GFAP (C) and HSPB1 (D) in areas proximal or distal to the plaques. *N* = 5 Braak V/VI cases, with a minimum of 10 images per case per condition. (**E**) The percentage of HSPB1^+^ cells that were also GFAP^+^ was calculated in areas that are proximal or distal to the plaques. *N* = 5 Braak V/VI cases. (**F**) Summary of literature review of the existing proteomic studies of CSF from healthy individuals, showing the total proteins detected, the number of samples tested, and the sHSPs identified. (**G**) (From left to right) Representative image of astrocytes in distal or proximal areas to plaques and the area selected for analysis (comprising an area of 50-μm diameter around nuclei); merged GFAP and HSPB1; and HSPB1 immunoreactivity in selected area; a GFAP mask is applied to distinguish between intracellular (GFAP^+^) and extracellular (GFAP^−^) space; and HSPB1 signal is shown after applying the GFAP mask, which shows the area in green used for quantification (non-masked HSPB1). (**H**) The mean fluorescence intensity of HSPB1 in the extracellular space (non-masked HSPB1) was calculated per astrocyte in distal or proximal regions to plaques. *N* = 5 Braak V/VI cases (>50 cells per case per condition). Data are shown as means ± SD. Paired Student’s *t* test (*n* = 5), *P < 0.05, ***P < 0.001.

To gain insights into HSPB1 in AD, we examined its cellular localization in the temporal cortex, where severe pathology develops at early stages. In human postmortem brains across different Braak stages of AD severity, HSPB1 is predominantly detected in astrocytes, as shown by its preferential location within cells positive for the general astrocyte marker aldehyde dehydrogenase 1 family member L1 (ALDH1L1) (Fig. 1A and fig. S1). HSPB1 protein was also expressed to a large extent in astrocytes positive for GFAP, widely used as marker of reactive astrocytes (Fig. 1A and fig. S1) (*2*). However, it was not detected in microglia (IBA1-positive) or oligodendrocytes (CAII-positive) and only occasionally in neurons (MAP2-positive) (Fig. 1A and fig. S1). In line with these findings, single-cell transcriptomic data of the sHSP family extracted from the Allen Brain Map showed that HSPB1 expression is limited to astrocytes and endothelial cells in control human brain and that its expression increases in astrocytes within AD progression (fig. S2). To better understand the relationship between HSPB1 and the astrocyte response to amyloid pathology in AD brain, we measured protein levels of HSPB1 within 50 μm from the edge of Aβ plaques, a distance that has been previously used to define plaque-associated local toxicity, including the accumulation of dystrophic neurites, activated microglia, and reactive astrocytes (*23*), which we referred to as “proximal” to plaques, in relation to equivalent areas beyond 50 μm from plaques, which were considered “distal” areas (Fig. 1B). The intensity of both GFAP and HSPB1 increased in the proximity of plaques (Fig. 1, C and D), and the proportion of HSPB1-expressing cells that were also GFAP-positive increased from 42.2% ± 6.4 in areas distal from plaques to 78.8% ± 8.5 in regions proximal to plaques (*P* = 0.0007) (Fig. 1E).

Amyloid plaques frequently showed positive staining for HSPB1 (fig. S3), as has been previously documented in AD brain (*24*), as well as in transgenic mouse models of AD (*25*), suggesting that HSPB1 may reach the extracellular space. A literature review of proteomic studies of human cerebrospinal fluid (CSF) (*26*–*40*) revealed that HSPB1 was identified in 12 of 14 studies that detected more than 1000 proteins in human CSF (Fig. 1F), supporting the notion that HSPB1 is not only restricted to intracellular compartments but also part of the extracellular milieu. Although less frequent, other sHSPs including CRYAB are also detected in human CSF (Fig. 1F). We reasoned that astrocytes are the source of extracellular HSPB1. Using GFAP to indicate the confines of astrocyte intracellular spaces, we could detect HSPB1 in the space surrounding astrocytes (non-masked HSPB1) (Fig. 1G). Moreover, quantification showed that extracellular HSPB1 was significantly higher around the astrocytes that are proximal to plaques compared to the astrocytes in distal regions (Fig. 1H). Our data show that HSPB1 expression is predominantly restricted to astrocytes in human brain. HSPB1 levels are higher in those reactive astrocytes that cluster around plaques in AD brain, as well as in their adjacent extracellular region, which, together with evidence collected from CSF studies, suggest that HSPB1 can be present in the extracellular space.

### Inflammatory reactive astrocytes secrete HSPB1

**Fig. 2.**
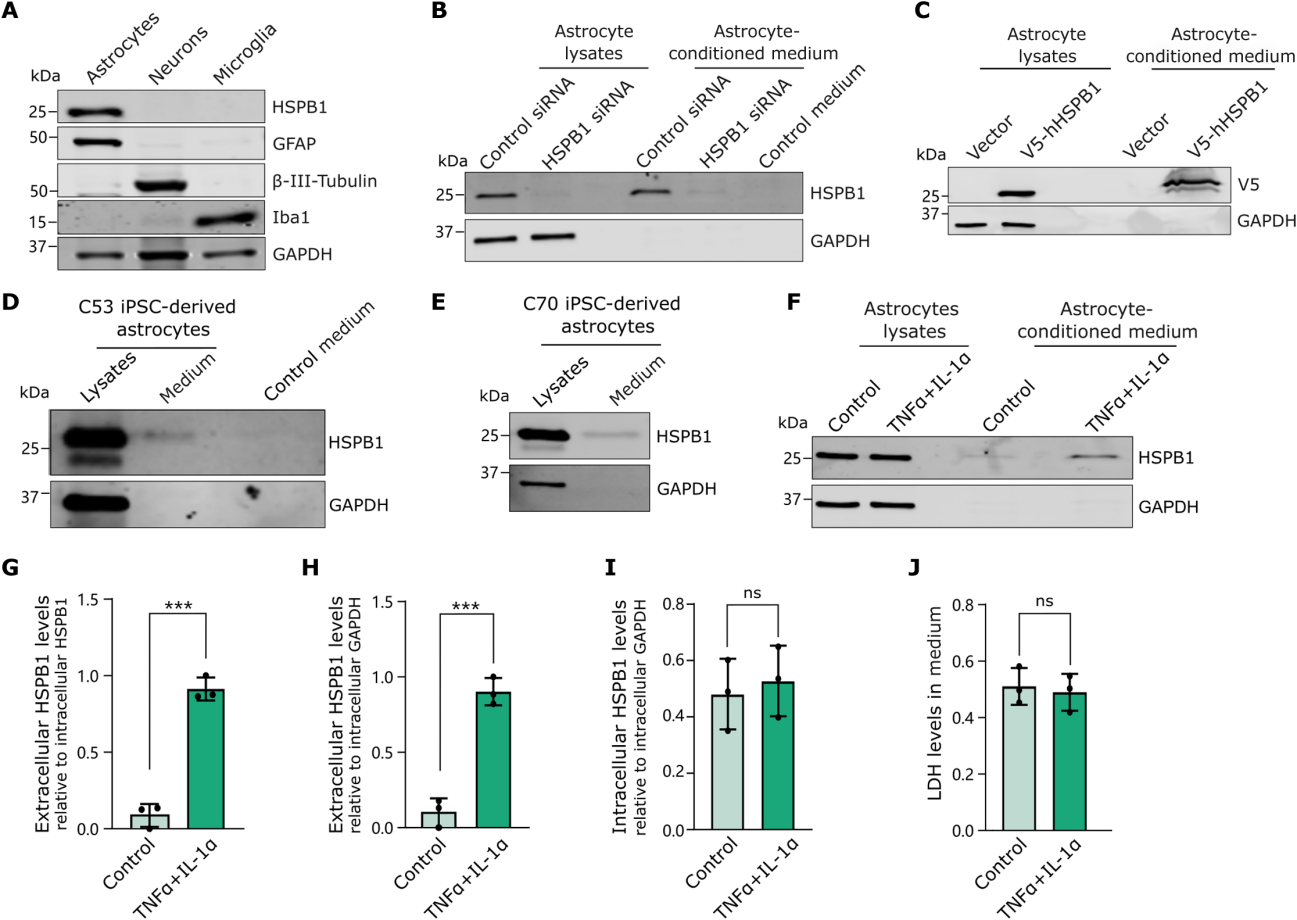
HSPB1 secretion is increased in inflammatory reactive astrocytes. (**A**) Levels of HSPB1 were detected by immunoblot in primary mouse astrocytes (GFAP) but not in neurons (β-III-tubulin) or microglia (IBA1). (**B**) Primary mouse astrocytes were treated with 50 nM scramble (control) or *HSPB1* siRNA, and HSPB1 was detected in astrocyte lysates or in concentrated conditioned medium. Fresh culture medium (non–cell exposed) was used as a control to discard the presence of HSPB1 or unspecific binding in serum. (**C**) Mouse astrocytes were transfected with either an empty vector or a construct to express V5-hHSPB1, which was detected by Western blotting with a V5 antibody in either astrocyte lysates or concentrated conditioned medium. (**D** and **E**) HSPB1 was detected in iPSC-derived astrocyte total cell lysates and conditioned medium in two control lines: C53 (D) and C70 (E). (**F** to **I**) Primary mouse astrocytes were treated with TNFα (30 ng/ml) and IL-1α (3 ng/ml) for 24 hours. (F) HSPB1 in lysates or in concentrated medium was detected by Western blotting, and levels of HSPB1 in medium were quantified relative to intracellular HSPB1 (G) or to intracellular GAPDH (H); intracellular HSPB1 levels were quantified relative to GAPDH (I); and LDH release to the medium was determined as a readout of cellular toxicity (**J**). Data are shown as means ± SD and were analyzed by unpaired Student’s *t* test in a minimum of three biological replicates. ***P < 0.001; ns, not significant.

To investigate the conditions driving HSPB1 secretion, we used mouse primary cultures where, as in human brain, expression of HSPB1 is detected in astrocytes but not in neurons or microglia (Fig. 2A). HSPB1 was also present in the astrocyte-conditioned medium of cultured mouse astrocytes, but not when astrocytes were treated with small interfering RNA (siRNA) to rule out any contamination from proteins in the serum (Fig. 2B). Moreover, human HSPB1 was also found in the medium of mouse astrocytes overexpressing V5-tagged human HSPB1 (V5-hHSPB1) (Fig. 2C). To extend these findings to human cells, we generated induced pluripotent stem cell (iPSC)–derived astrocytes following a small-molecule induction protocol and treatment with ciliary neurotrophic factor (CNTF) for 14 days to yield mature astrocytes, as described by Serio *et al.* (*41*) (fig. S4). HSPB1 expression was confirmed in mature astrocytes from two healthy control iPSC lines (Fig. 2, D and E, and fig. S4), as well as in their conditioned medium (Fig. 2, D and E). These data confirm that HSPB1 is secreted from mouse and human astrocytes.

Our data from human brain suggest that HSPB1 secretion increases in GFAP-reactive astrocytes associated with amyloid plaques. Astrocyte response is modulated by exposure to signals from other cells, including those released from activated microglia (*3*), which can be modeled by exposing astrocytes to tumor necrosis factor α (TNFα) and interleukin-1α (IL-1α) (*3*). Treatment of murine primary astrocytes with these cytokines caused an increase in markers of astrocyte reactivity, such as LCN2 and SerpinA3N (fig. S5, A to C) (*2*), which was accompanied by an enhanced expression of inducible nitric oxide synthase (iNOS) and increased secretion of nitric oxide (NO) to the medium (fig. S5, D to F). Treatment with TNFα + IL-1α also induced the translocation of the p65 subunit of the transcription factor nuclear factor κB (NF-κB) into the nucleus, consistent with the induction of NF-κB–mediated inflammatory signaling pathways (fig. S5G), together with increased secretion of the cytokine IL-6 (fig. S5H). This response is comparable to that displayed by astrocytes cultured with medium from activated microglia that had been exposed to lipopolysaccharide (LPS) and interferon γ (IFNγ) (fig. S6, A to F), indicating that treatment with TNFα + IL-1α recapitulates the astrocyte inflammatory reaction in response to activated microglia. We then investigated HSPB1 secretion from inflammatory reactive astrocytes stimulated with TNFα + IL-1α, which resulted in a marked increase in the levels of endogenous HSPB1 in the extracellular medium (Fig. 2, F to H), as well as secretion of human HSPB1 in astrocytes overexpressing V5-hHSPB1 (fig. S5, J to L). This cannot be explained by increased expression of HSPB1 (Fig. 2, F and I, and figs. S5I and S6, G and H) or by cell toxicity (Fig. 2J). A significant increase in HSPB1 phosphorylation, known to regulate its oligomerization and activity (*5*), was observed in reactive astrocytes induced with either treatment of cytokines or medium from activated microglia (fig. S5, M and N). Our findings demonstrate that, in line with data from human AD brain, inflammatory reactive astrocytes increase the secretion of HSPB1, which may potentially be regulated by changes in its phosphorylation status.

### HSPB1 is secreted as a free protein, not contained within EVs

**Fig. 3.**
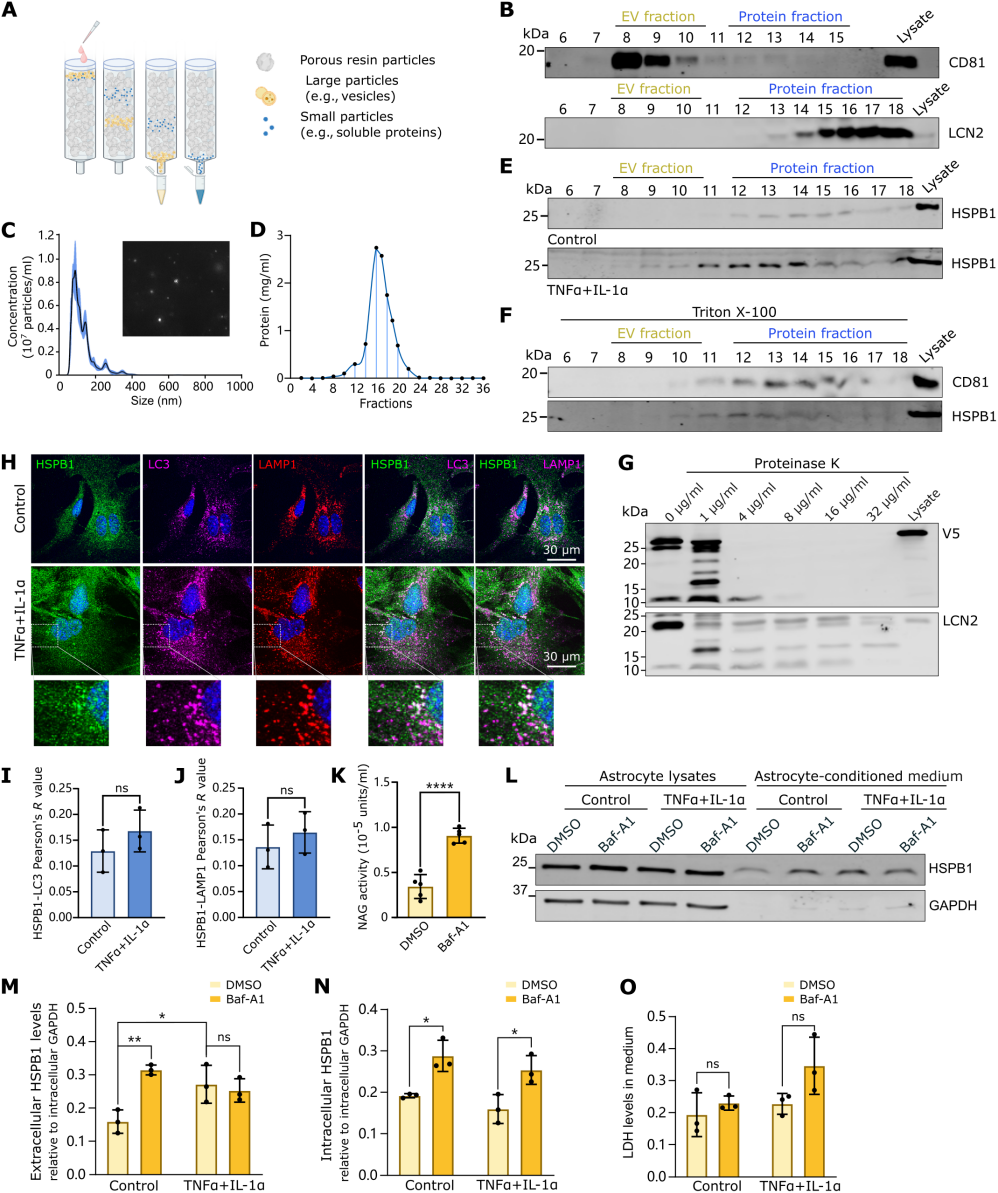
HSPB1 is not secreted within EVs. (**A**) SEC was used to fractionate the medium and to separate large particles such as EVs from soluble free proteins. (**B**) Astrocyte-conditioned medium was fractionated by SEC, and fractions were immunoblotted for CD81 (EV marker) and LCN2 (secreted as free protein). (**C**) The concentration and size distribution of particles contained in fractions 7 to 9 were analyzed by NTA. (**D**) Total protein concentration was determined by NanoDrop spectrophotometer in fractions 1 to 36. (**E**) Conditioned medium from astrocytes treated with control or TNFα + IL-1α for 24 hours was fractionated by SEC and immunoblotted for HSPB1. (**F**) Astrocyte medium was treated with 1% Triton X-100 for 1 hour before SEC, followed by detection of CD81 and HSPB1. (**G**) Medium of astrocytes expressing V5-hHSPB1 was treated with increasing concentrations of proteinase K, and levels of HSPB1 and LCN2 and bands resulting from its degradation were detected by Western blotting. (**H**) HSPB1, LAMP1, and LC3 were detected by immunofluorescence in astrocytes treated with control or TNFα + IL-1α for 24 hours, and colocalization was determined as Pearson’s correlation coefficient for HSPB1-LC3 (**I**) and HSPB1-LAMP1 (**J**). (**K** to **O**) Astrocytes were treated with dimethyl sulfoxide (DMSO) (control) or 20 nM Baf-A1 for 24 hours: Levels of NAG were detected in the medium (K); HSPB1 was detected in lysates or concentrated medium after concomitant treatment with control or TNFα + IL-1α and Baf-A1 (L), and the levels of HSPB1 in medium (M) or the levels of intracellular HSPB1 (N) were quantified relative to GADPH in lysates; and LDH levels in medium relative to intracellular levels (O). Data are shown as means ± SD and analyzed by two-way ANOVA with Tukey’s multiple comparisons test [(M) to (O)] or unpaired Student’s *t* test [(I) to (K)] in a minimum of three biological replicates. *P < 0.05, **P < 0.01, ***P < 0.001.

HSPB1 does not contain a signal peptide, which prompted us to investigate its release through alternative mechanisms, such as secretion within extracellular vesicles (EVs), where HSPB1 has been previously detected (*42*–*44*). Size exclusion chromatography (SEC) (Fig. 3A) was used to separate astrocyte-conditioned medium into fractions that are enriched in EVs (fractions 8 to 10). These fractions are characterized by the presence of the tetraspanin CD81 (Fig. 3B) and for containing particles of 60 to 150 nm, as detected by nanoparticle tracking analysis (NTA), which are compatible with small EVs, such as exosomes (Fig. 3C) (*45*). Total free protein eluted in fractions 12 to 21 (Fig. 3D) and, as expected, proteins secreted through conventional secretion such as LCN2 (*46*, *47*) were found in these fractions (Fig. 3B). Unexpectedly, HSPB1 did not elute in the EV fractions but peaked in fractions 12 to 14, suggesting its secretion as a free protein (Fig. 3E). While HSPB1 levels were higher in the medium upon treatment with cytokines, its elution pattern was largely similar, indicating that HSPB1 secretion is increased from inflammatory reactive astrocytes, but its secretion route is not altered (Fig. 3E). To further confirm that HSPB1 was not secreted within vesicles of smaller size that may elute at later fractions, astrocyte-conditioned medium was treated with Triton X-100 before fractionation or with proteinase K. Upon membrane disruption with Triton X-100, CD81 shifted from the EV fraction to the total protein fraction, while HSPB1 elution remained in the same fractions (Fig. 3F). Incubation of medium from astrocytes expressing V5-hHSPB1 with increasing concentrations of proteinase K led to the degradation of HSPB1 as well as degradation of the non-EV protein LCN2 (Fig. 3G). Together, these data confirm that astrocytic HSPB1 is not primarily secreted within EVs. Moreover, TNFα + IL-1α treatment did not induce changes in HSPB1 secretion pattern or in the secretion of the EV marker CD81 (fig. S7).

Alternative secretion mechanisms include the autophagosome-lysosomal system, previously implicated in the secretion of HSPB1 from macrophages (*48*) or endothelial cells (*49*). Moreover, it has recently been suggested that treatment with proinflammatory cytokines leads to increased lysosomal secretion in inflammatory reactive astrocytes (*50*). In line with these findings, we observed partial colocalization of HSPB1 with the autophagosome marker LC3 and with the lysosomal marker LAMP1 (Fig. 3H) and, while not significant, this colocalization tended to increase in inflammatory reactive astrocytes treated with TNFα + IL-1α compared to control astrocytes (Fig. 3, I and J). To further validate whether lysosomes may act as reservoirs from which HSPB1 is secreted into the medium, we induced lysosomal secretion by treating astrocytes with low doses of bafilomycin A1 (Baf-A1) (20 nM) that induces lysosomal exocytosis (*50*, *51*), including the secretion of the soluble lysosomal enzyme *N*-acetyl-β-d-glucosaminidase (NAG) (Fig. 3K), which was increased in parallel with elevated HSPB1 in the medium (Fig. 3, L and M) but did not increase cellular toxicity (Fig. 3O). This effect did not further exacerbate the secretion of HSPB1 induced by TNFα + IL-1α, suggesting that both treatments act upon similar secretory mechanisms (Fig. 3, L and M). While double treatment seems to be slightly toxic, this effect was not significant. Furthermore, it cannot explain changes in HSPB1 secretion, since this would result in enhanced detection of extracellular HSPB1 upon combined treatment (Fig. 3O). Despite the low concentrations of Baf-A1 used, intracellular levels of HSPB1 also increased (Fig. 3N), suggesting that HSPB1 may be a substrate for the autophagy lysosomal pathway, and we can hypothesize that it can be targeted for either degradation or secretion upon different stimuli. Together, our findings show that HSPB1 is primarily secreted as a free protein that is not contained within EVs.

### Astrocytes and neurons uptake astrocyte-secreted HSPB1

**Fig. 4.**
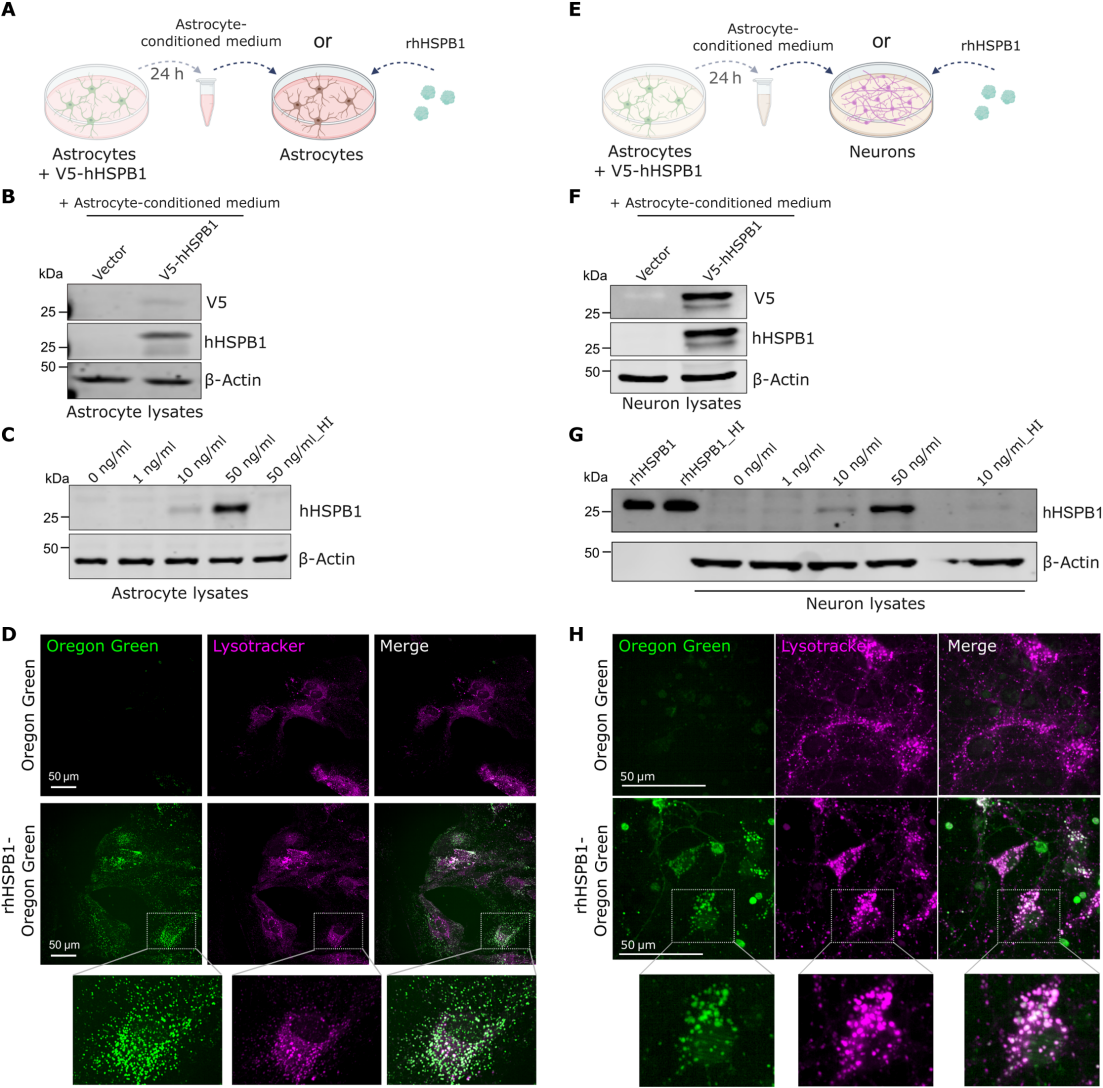
HSPB1 is internalized by astrocytes and neurons. (**A**) Primary mouse astrocytes were treated for 24 hours either with conditioned medium from astrocytes transfected with V5-hHSPB1 or with rhHSPB1. Created with BioRender.com. (**B**) Anti-V5 and anti-human HSPB1 (hHSPB1) antibodies were used to detect the levels of V5-hHSPB1 in astrocyte lysates exposed to conditioned medium for 24 hours. (**C**) An anti-hHSPB1 antibody was used to detect the levels of human HSPB1 in astrocytes treated with the indicated concentrations of rhHSPB1 for 24 hours or with heat-inactivated (HI) rhHSPB1. (**D**) Confocal live imaging of astrocytes exposed to rhHSPB1 labeled with Oregon Green for 24 hours and stained with lysotracker. (**E**) Primary mouse neurons were treated with either conditioned medium from astrocytes transfected with V5-hHSPB1 and grown in Neurobasal medium or with rhHSPB1. Created with BioRender.com. (**F**) Anti-V5 and anti-hHSPB1 antibodies were used to detect the levels of V5-hHSPB1 in neuron lysates exposed to conditioned medium for 8 hours. (**G**) An anti-hHSPB1 antibody was used to detect the levels of human HSPB1 in neurons treated with the indicated concentrations of rhHSPB1, or heat-inactivated rhHSPB1, for 24 hours. Equivalent amounts of rhHSPB1 in control or upon heat inactivation were run in parallel to confirm its detection by Western blotting. Note that the background detected in neuron lysates at 0 ng/ml is unspecific since (i) neurons in culture do not express HSPB1, as we have shown in Fig. 2A, and (ii) human HSPB1 antibody does not recognize the mouse HSPB1 [as shown in (C), where astrocytes express high levels of murine HSPB1]. (**H**) Confocal live imaging of neurons exposed for 6 hours to rhHSPB1 labeled with Oregon Green and stained with lysotracker.

To further elucidate the fate of secreted HSPB1, astrocytes were transfected with V5-hHSPB1 or empty vector, and conditioned medium was collected after 24 hours and used to culture untransfected astrocytes for a further 24 hours (Fig. 4A). Using antibodies against V5 or that specifically target human HSPB1, we could detect human HSPB1 in the lysates of astrocytes exposed to V5-hHSPB1–containing medium (Fig. 4B). To further confirm that astrocytes uptake HSPB1 that is free in the extracellular medium, we next treated astrocytes with increasing concentrations of rhHSPB1. To mimic the physiological concentrations and to avoid overloading with exogenous protein, we used concentrations within the nanogram per milliliter range, which is found in the astrocyte-conditioned medium (fig. S8) and in human CSF (*52*). Exposure of astrocytes to rhHSPB1 resulted in its internalization by astrocytes, but this was prevented when the recombinant protein was heat-inactivated, confirming that internalization requires an intact protein conformation (Fig. 4C). Fluorescent labeling of rhHSPB1 with Oregon Green was then used to explore the subcellular localization of the internalized HSPB1, which showed a punctate staining in the soma of astrocytes that overlapped with lysotracker-positive vesicles (Fig. 4D).

Next, we explored whether the internalization of extracellular hHSPB1 is limited to astrocytes or whether it extends to other cell types. As done for astrocytes, primary mouse neurons were exposed to medium from astrocytes that have been transfected with V5-hHSPB1 (Fig. 4E). After 8 hours, V5-hHSPB1 was detected in the neuron lysates (Fig. 4F). Similar findings were observed when neurons were treated with exogenously added rhHSPB1 but not when this was heat-inactivated (Fig. 4G). Likewise, in neurons exposed to rhHSPB1–Oregon Green, it appeared as puncta that were also lysotracker positive (Fig. 4H). While these were mostly found in the soma, some could also be found in the neuronal projections. Our findings reveal that astrocytes and neurons can uptake astrocyte-secreted HSPB1. Uptake of hHSPB1 seems to follow similar routes in both astrocytes and neurons, being directed to acidic vesicles, compatible with lysosomes and late endosomes, and suggesting its internalization via endocytosis mechanisms. While our data in human brain show that HSPB1 is detected mainly in astrocytes, these findings could potentially explain that some neurons are also positive for HSPB1.

### Extracellular HSPB1 dampens the inflammatory response in astrocyte cultures and in an ex vivo model

**Fig. 5.**
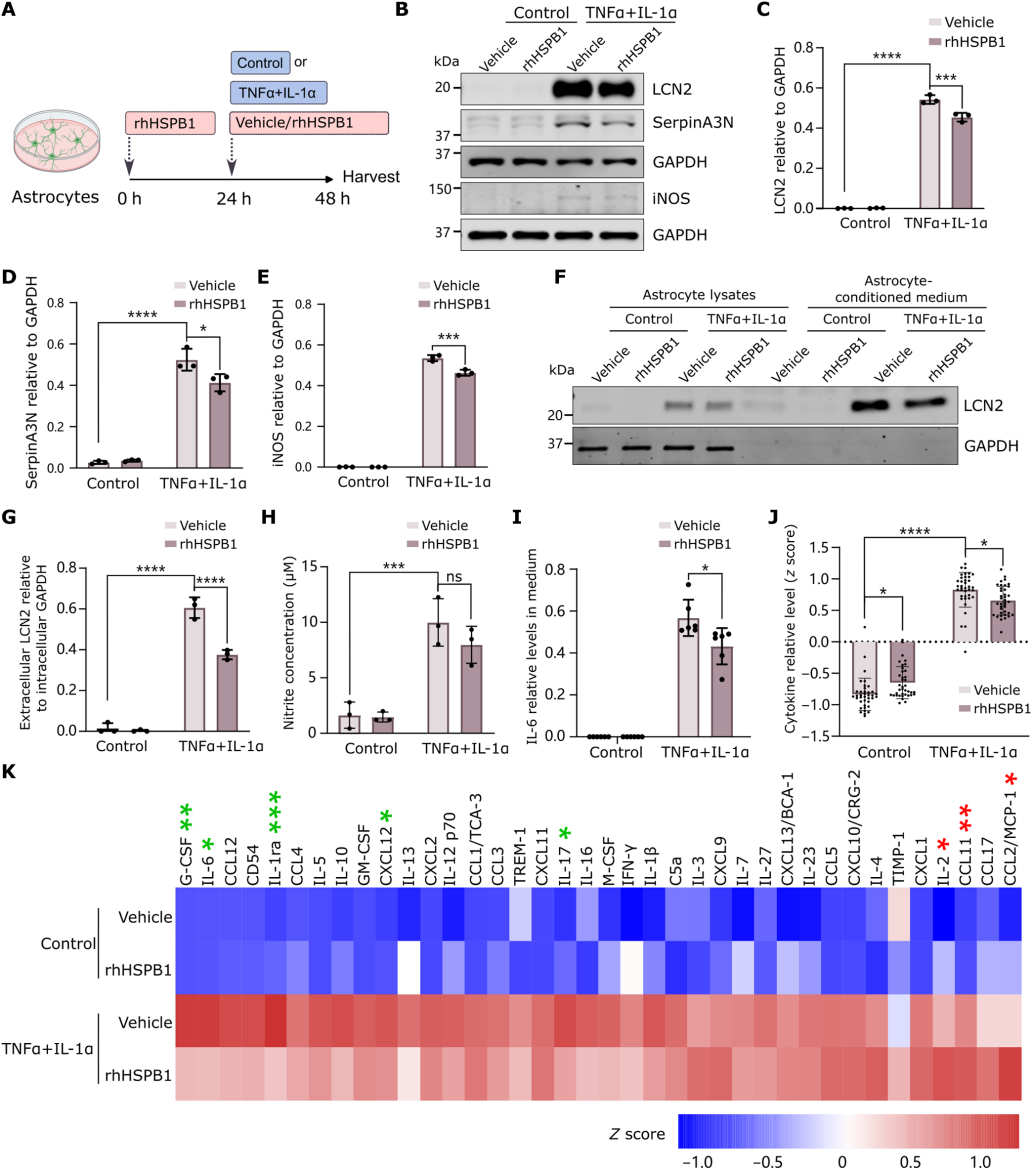
rhHSPB1 attenuates the astrocyte inflammatory reactive response. (**A**) Primary astrocytes were pretreated with rhHSPB1 for 24 hours before treatment with either 0.1% BSA (vehicle) or rhHSPB1 (50 ng/ml) and either TNFα + IL-1α or control for further 24 hours. Created with BioRender.com. (**B**) LCN2, SerpinA3N, and iNOS were detected by Western blotting in astrocyte lysates, and levels were quantified relative to GADPH as a loading control (**C** to **E**). (**F** and **G**) LCN2 was detected in concentrated conditioned medium and quantified relative to intracellular GAPDH levels (G). (**H**) The concentration of nitrites in the astrocyte medium was measured as an indirect measure of NO using a Griess assay. (**I**) The levels of IL-6 in the astrocyte medium were determined by ELISA. (**J** and **K**) A cytokine array was used to determine levels of 38 cytokines in the medium in a membrane-based sandwich immunoassay. The average *z* scores for each of the 38 cytokines was represented in the graph (J) or shown individually for each cytokine in the heatmap (K), where cytokines are ranked from low to high fold change of TNFα + IL-1α/rhHSPB1 versus TNFα + IL-1α/vehicle. Cytokines with an asterisk showed significant increase (red) or decrease (green) in TNFα + IL-1α/rhHSPB1 versus TNFα + IL-1α/vehicle condition. Data are shown as means ± SD (*N* = 3). Two-way ANOVA with Tukey’s or Sidak’s multiple comparisons test [(C), (D), (E), (G), (H), and (J)] or unpaired Student’s *t* test (I). *P < 0.05, **P < 0.01, ***P < 0.001, ****P < 0.0001.

Next, we explored the effect that extracellular HSPB1 may have on astrocytes. Because sHSPs, and in particular HSPB1, have been shown to modulate the inflammatory response (*18*), we questioned whether secreted HSPB1 may also have a role in modulating astrocyte reactivity. As we have demonstrated that HSPB1 is secreted as a free protein and that both astrocyte-derived and recombinant protein can be internalized in a similar manner, we used rhHSPB1 to explore this effect in a controlled environment. Pretreatment of cultured astrocytes with rhHSPB1 followed by an induction of inflammatory reactive astrocytes with TNFα + IL-1α resulted in a significant reduction in the astrocyte reaction, including lower levels of intracellular LCN2, SerpinA3N, and iNOS (Fig. 5, B and E), an effect that was not observed when treating with heat-inactivated rhHSPB1 (fig. S9). Concomitantly, we observed that treatment with rhHSPB1 reduced secretion of the toxic and proinflammatory factors LCN2 (Fig. 5, F and G) and, albeit not significant, reduced NO (Fig. 5H), and significantly reduced secretion of the cytokine IL-6 (Fig. 5I). Treatment with TNFα + IL-1α for 24 hours resulted in an increase in cytokine and chemokine secretion as detected by an antibody-based array (Fig. 5, J and K, and fig. S10A), and exposure to rhHSPB1 resulted in an overall decrease of this response (Fig. 5, J and K), with significant reductions in IL-17, IL-6, CXCL12, IL-1ra, and granulocyte colony-stimulating factor (G-CSF) and increases in CCL2, CCL11, and IL-2, compared to vehicle-treated cells (Fig. 5K and fig. S10, B to I). Our data show that treatment with rhHSPB1 ameliorates the reactive inflammatory response in primary mouse astrocytes as shown by reduced markers of astrocyte reactivity and reduced secretion of mediators of neurotoxicity and inflammation such as LCN2 (*46*, *47*) and an overall decrease in cytokine secretion.

**Fig. 6.**
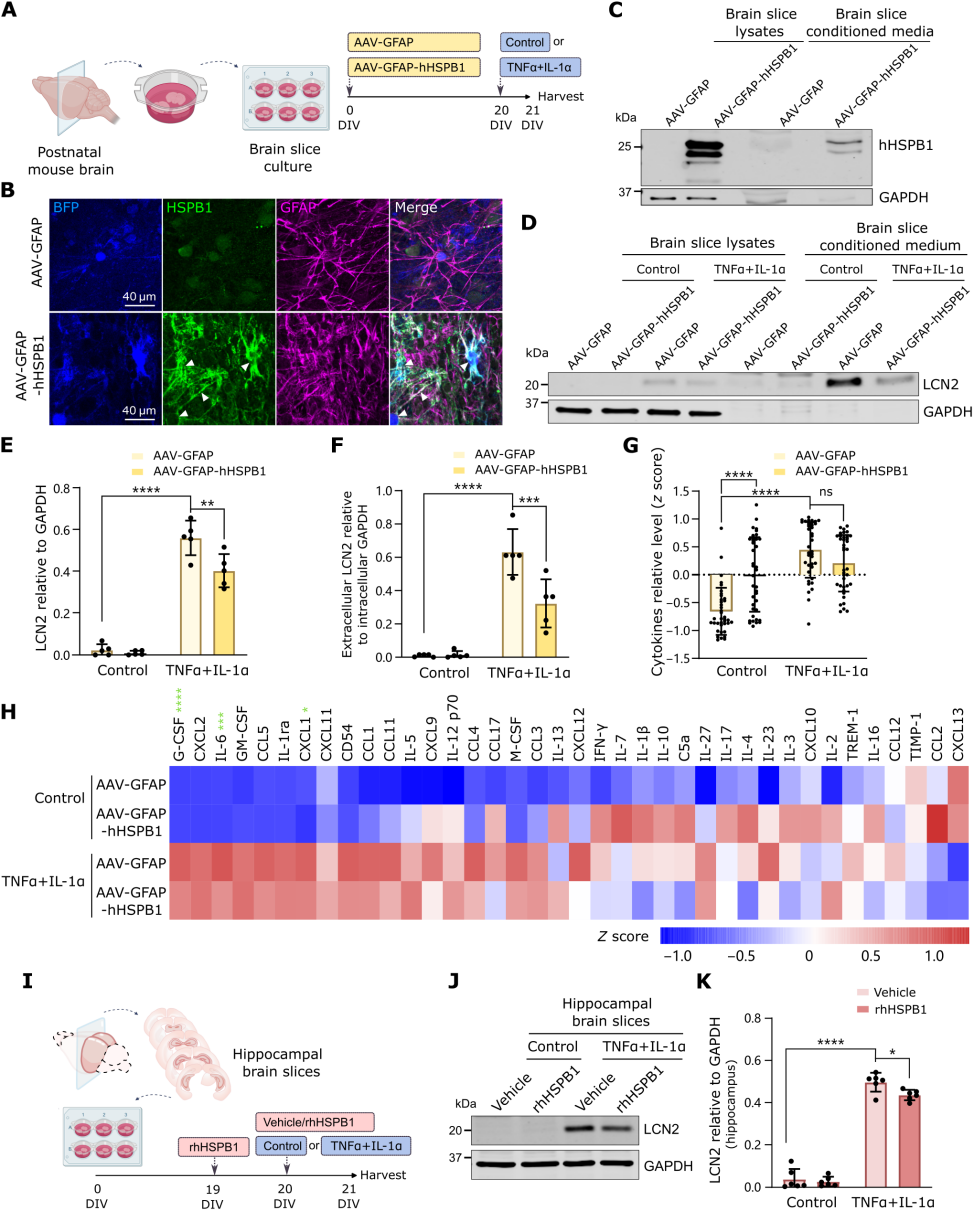
Astrocyte-secreted HSPB1 modulates the inflammatory response in organotypic brain slice cultures. (**A**) Organotypic brain slices were prepared from postnatal mouse brain, transduced at 0 DIV with AAVs to express hHSPB1 in astrocytes under the GFAP promoter for 20 DIV followed by treatment with TNFα + IL-1α for 24 hours. Created with BioRender.com. (**B**) Transduction of slices with AAV-GFAP-BFP (AAV-GFAP) and AAV-GFAP-hHSPB1:BFP (AAV-GFAP-hHSPB1) shows astrocyte-specific (GFAP^+^) expression of BFP and hHSPB1 (note that HSPB1 antibody has some unspecific nuclear staining). (**C**) Western blotting to confirm expression of hHSPB1 and its secretion in brain slices. (**D** to **F**) Changes in LCN2 were quantified in lysates (E) and in conditioned medium (F) relative to intracellular GAPDH. (**G** and **H**). A cytokine array measured levels of 38 cytokines in the medium in a membrane-based sandwich immunoassay. The average *z* scores for each cytokine were represented in the graph (G) or shown individually in the heatmap (H), where cytokines are ranked from high to low fold change of control/AAV-GFAP versus TNFα + IL-1α/AAV-GFAP. Cytokines marked with a green asterisk were significantly decreased in TNFα + IL-1α/AAV-GFAP-hHSPB1 versus TNFα + IL-1α/AAV-GFAP conditions. (**I**) The hippocampus was separated and cultured for 19 days and then pretreated with rhHSPB1 (50 ng/ml) for 24 hours and with either control or TNFα + IL-1α and either vehicle (0.1% BSA) or rhHSPB1 (50 ng/ml) for further 24 hours. (**J**) Levels of LCN2 were detected by immunoblot in slice lysates and quantified relative to GAPDH (**K**). Data are shown as means ± SD. *N* = 5 [(E) and (F)], *N* = 3 [(G) and (H)], or *N* = 6 [(J) and (K)]. Two-way ANOVA with Tukey’s or Sidak’s multiple comparisons test, *P < 0.05, **P < 0.01, ***P < 0.001, ****P < 0.0001.

We next used organotypic brain slice cultures as an ex vivo brain model where neuronal and glial cells coexist and brain architecture is preserved and that can recapitulate disease features in an accelerated timescale (Fig. 6A) (*53*). Expression of human HSPB1 under a short form of the GFAP promoter and its packaging into adeno-associated viruses (AAVs) with the 2/8 serotype (fig. S11) (*54*) allows limiting of its expression to a subset of astrocytes in brain slices, as detected by hHSPB1 and blue fluorescent protein (BFP) (cotranslated with hHSPB1) signal in GFAP-positive cells (Fig. 6B). hHSPB1 was detected in lysates as well as in the medium (Fig. 6C), providing a valuable tool to assess the effect of astrocyte-secreted HSPB1 in brain slices. Treatment with proinflammatory cytokines TNFα + IL-1α for 24 hours led to a response that resembled that of primary astrocytes in culture, with an increase in intracellular and secreted LCN2 (Fig. 6, D to F). In line with our previous data, changes in LCN2 upon inflammatory stimulation were significantly reduced with AAV-GFAP-hHSPB1 compared to AAV-GFAP control (Fig. 6, D to F). Although less apparent than in cultured astrocytes, treatment with TNFα + IL-1α led to a general increased secretion of cytokines and chemokines in brain slices, which was overall reduced with astrocytic hHSPB1 (Fig. 6, G and H, and fig. S10J), and which was significant for G-CSF, IL-6, and CXCL1 (Fig. 6H and fig. S10, K to M). In control conditions, expression of hHSPB1 induced secretion of a number of cytokines, in line with what we also observed in primary astrocytes, but in most cases, this effect was the opposite when slices were also treated with TNFα + IL-1α (Fig. 6H). To confirm that this is a result of extracellular HSPB1, we treated brain slices with rhHSPB1 or vehicle control, together with TNFα + IL-1α. Because the effect of AAV-GFAP-hHSPB1 was more pronounced in coronal slices from posterior brain regions that are enriched in hippocampus, compared to slices from the anterior brain (fig. S12, A to C), we assessed the effect of rhHSPB1 in slices from posterior regions, which resulted in a significant reduction in intracellular and secreted levels of LCN2 (fig. S12, D to F) similar to the effect observed in slices prepared from hippocampus (Fig. 6, I to K). Combined data from cultured astrocytes and organotypic brain slices show that the presence of HSPB1 in the extracellular environment diminishes the inflammatory reaction in astrocytes.

### Extracellular HSPB1 confers neuronal protection and reduces tau aggregate burden in neurons

**Fig. 7.**
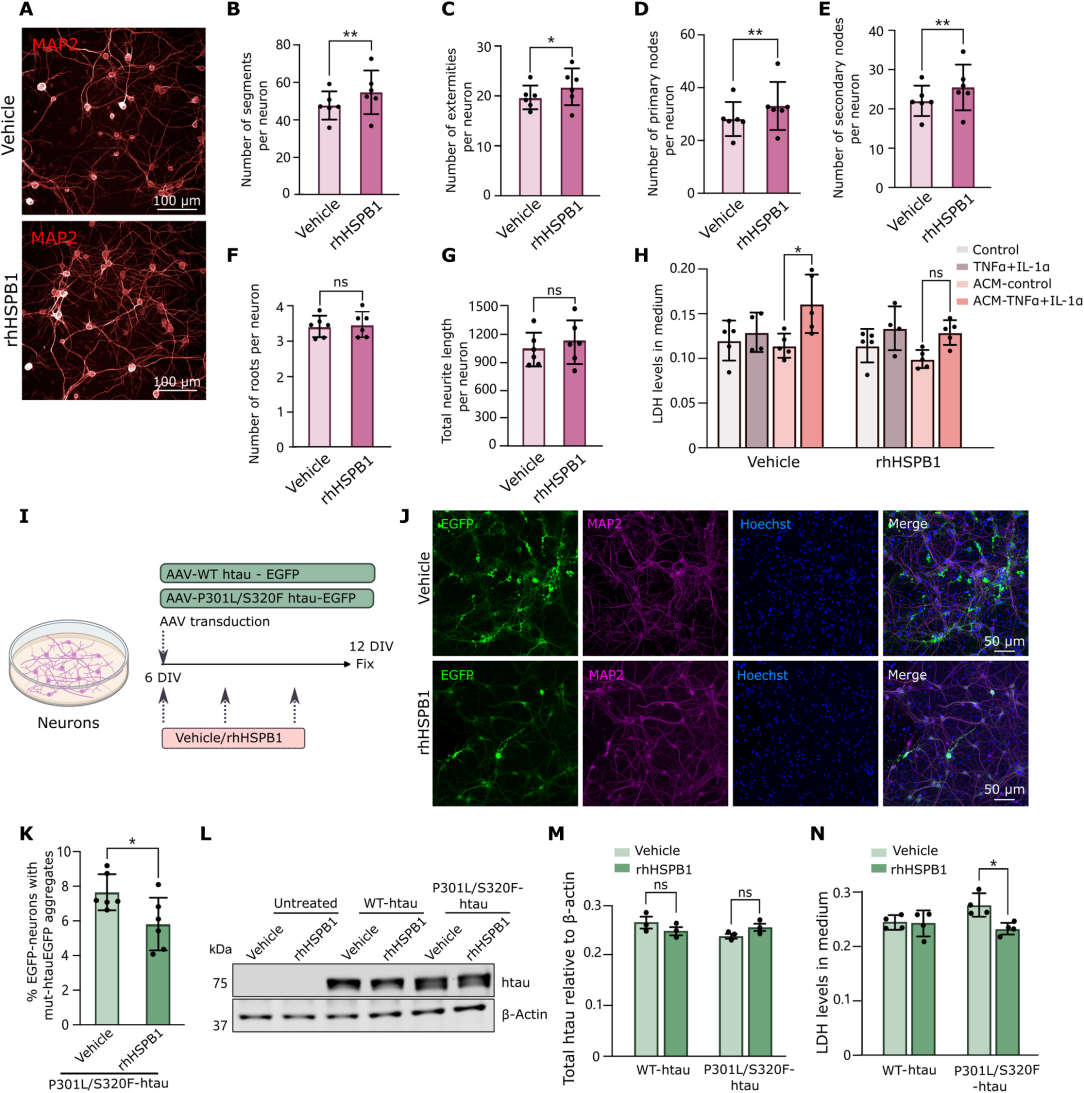
Extracellular hHSPB1 promotes neuronal health and prevents human mutant tau aggregation in primary mouse neurons. (**A**) Primary mouse neurons were treated with rhHSPB1 (10 ng/ml) or vehicle (0.1% BSA) every 3 days from 3 DIV, fixed on 14 DIV, and stained for MAP2. (**B** to **G**) High-content confocal microscopy was used to determine changes in the number of segments (B), end points (C), primary branching points (D), secondary branching points (E), root points (F) per neuron, and the total length of all neurites for each neuron (G) (*N* = 6). (**H**) Neurons were treated with conditioned medium from control or TNFα + IL-1α–treated astrocytes at 10 and 12 DIV, together with rhHSPB1 (50 ng/ml) or vehicle (0.1% BSA), and the medium was collected at 13 DIV. In parallel, neurons were treated with equivalent concentrations of TNFα + IL-1α. Cellular toxicity was determined by measuring LDH levels in neuron-conditioned medium (*N* = 5). (**I**) Neurons were transduced at 6 DIV with AAVs to express WT-htau-EGFP or mutant P301L/S320F-htau-EGFP and treated with either vehicle (0.1% BSA) or rhHSPB1 (50 ng/ml) every 2 days and fixed at 12 DIV. Created with BioRender.com. (**J**) Representative images of neurons transduced with mutant P301L/S320F-htau-EGFP and treated with vehicle (0.1% BSA) or rhHSPB1 (50 ng/ml). (**K**) Graph shows the percentage of EGFP-positive neurons that show mutant htau aggregates. *N* = 6 with >1500 cells counted per experiment. (**L** and **M**) Total levels of tau were detected using an antibody that recognizes total tau, and the band corresponding to htau tagged to EGFP was quantified relative to β-actin. (**N**) Cellular toxicity was determined by measuring LDH levels in neuron-conditioned medium (*N* = 4). Data are shown as means ± SD. Paired Student’s *t* test [(B) to (G) and (K)]; two-way ANOVA with Tukey’s multiple comparisons test [(H), (M), and (N)], *P < 0.05, **P < 0.01.

To further explore the role of secreted HSPB1, we treated primary mouse neurons with rhHSPB1 every 3 days up to 14 days in vitro (DIV), followed by staining of neuronal projections with a MAP2 antibody (Fig. 7A). This resulted in an increase in parameters indicative of neuronal branching in response to treatment with extracellular human HSPB1 (Fig. 7, B to F), while no changes were observed in the length of the neurites (Fig. 7G). Incubation of neurons with conditioned medium from reactive astrocytes for 3 days led to an expected increase in neuronal toxicity (*3*), as measured by the release of lactate dehydrogenase (LDH) to the medium (Fig. 7H). This toxicity was partially abolished in neurons treated with rhHSPB1, supporting a neuroprotective function of extracellular human HSPB1.

To investigate whether extracellular HSPB1 modulates tau inclusion pathology in neurons, we used AAVs to express enhanced green fluorescent protein (EGFP)–tagged human tau (htau) bearing the P301L and S320F mutations in *MAPT*, which has propensity to aggregate (*54*). Transduction of neurons at 6 DIV with viral vectors expressing P301L/S320F-htau-EGFP resulted in the formation of aggregate-like structures at 12 DIV (Fig. 7, I and J) that were positively labeled with the MC1 antibody, which detects tau in an abnormal conformation (fig. S13A). The proportion of neurons with P301L/S320F-htau-EGFP aggregates was significantly reduced when neurons were repeatedly treated with rhHSPB1 (50 ng/ml) (Fig. 7, J and K), an effect that was lost when rhHSPB1 was heat-inactivated (fig. S14) and that does not depend on changes in the levels of total htau (Fig. 7, L and M). Moreover, treatment with rhHSPB1 reduced toxicity in neurons expressing mutant htau, as shown by reduced levels of LDH in the medium (Fig. 7N).

**Fig. 8.**
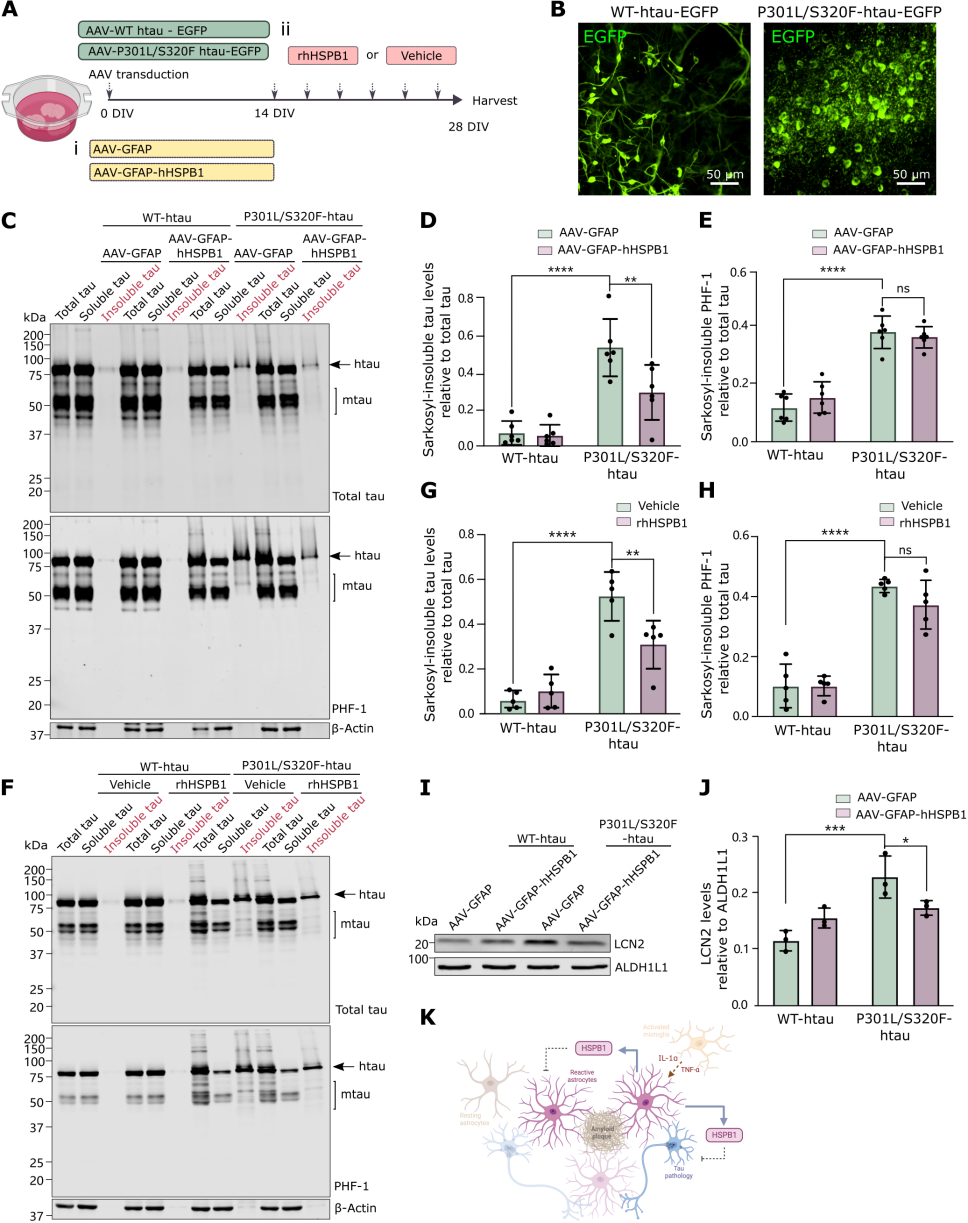
Extracellular hHSPB1 reduces accumulation of sarkosyl-insoluble P301L/S320F-htau in organotypic brain slices. (**A**) Organotypic brain slices were transduced with AAVs to express WT-htau-EGFP or mutant P301L/S320F-htau-EGFP and (i) simultaneously transduced with AAV-GFAP or AAV-GFAP-hHSPB1 or (ii) treated with either vehicle (0.1% BSA) or rhHSPB1 (50 ng/ml) from 14 DIV and then every 2 to 3 days up to 28 DIV. Created with BioRender.com. (**B**) Representative images of slices with AAV-WT-htau-EGFP or AAV-P301L/S320F-htau-EGFP. (**C** to **E**) Total lysates from AAV-GFAP-HSPB1 or control transduced slices were subjected to sarkosyl extraction to isolate the low-speed supernatant (total tau), high-speed supernatant (soluble tau), or sarkosyl-insoluble pellet (insoluble tau). Tau (D) or Ser^396^/Ser^404^ phosphorylated tau (PHF-1) (E) were quantified in the whole lane (between 15 and 250 kDa) to include both mouse (mtau) and htau and any modified tau, and quantified relative to β-actin or total tau, as indicated. (**F** to **H**) Lysates from brain slices treated with rhHSPB1 or vehicle were subjected to sarkosyl extraction, and levels of tau (G) or PHF-1 (H) were quantified as above. (**I**) Intracellular LCN2 was detected in lysates from slices transduced with AAV-WT-htau-EGFP or AAV-P301L/S320F-htau-EGFP and AAV-GFAP-HSPB1 or control at 0 DIV and collected at 28 DIV. Levels of LCN2 relative to ALDH1L1 are shown (**J**). Data are shown as means± SD. *N* = 6 [(D) and (E)], *N* = 5 [(G) and (H)], or *N* = 3 (J). Two-way ANOVA with Tukey’s multiple comparisons test [(D), (E), (G), (H), and (J)], *P < 0.05, **P < 0.01, ***P < 0.001, ****P < 0.0001. (**K**) Working model showing that reactive astrocytes that surround amyloid plaques or that are exposed to proinflammatory cytokines from activated microglia secrete HSPB1 into the extracellular space. Secreted HSPB1 can have autocrine functions by ameliorating the reactive inflammatory response in astrocytes, as well as paracrine functions by reducing tau inclusion pathology and promoting neuronal health in adjacent neurons. Created with BioRender.com.

The same AAVs were used to drive the neuronal expression of P301L/S320F-htau-EGFP in organotypic brain slice cultures. After 28 days in culture, this model develops inclusions in neurons that are positive for thioflavin S and that contain hyperphosphorylated, sarkosyl-insoluble htau at 75 kDa that can be distinguished from 50 to 55 kDa mouse tau (mtau) (Fig. 8, A to D, and fig. S13B) (*54*, *55*). First, we tested whether the increased expression of hHSPB1 in astrocytes is sufficient to lower the accumulation of aggregated tau (Fig. 8A). Transduction of slices with AAV-GFAP-hHSPB1 reduced the accumulation of sarkosyl-insoluble tau without affecting the levels of tau in the total or sarkosyl-soluble fraction (Fig. 8, C and D, and fig. S15, A to B). To further determine whether the effect of astrocytic hHSPB1 is mediated by secreted HSPB1, brain slices expressing either wild type or P301L/S320F-htau-EGFP were treated with rhHSPB1 (50 ng/ml) or vehicle from 14 DIV and every 2 to 3 days (Fig. 8A). In line with our previous observations, the presence of rhHSPB1 in the extracellular environment resulted in a significant decrease in the amount of mutant htau in the insoluble fraction (Fig. 8, F and G), with no variations in tau in the total or soluble fractions (fig. S15, E and F). These reductions in insoluble tau are not accompanied by significant changes in tau phosphorylation in residues S396/404 (PHF-1) (Fig. 8, C, E, F, and H, and fig. S15, C, D, G, and H). Expression of P301L/S320F-htau-EGFP in brain slices led to an increase in the proinflammatory factor LCN2, and in line with our previous findings, AAV-GFAP-hHSPB1 was also able to partially revert this effect (Fig. 8, I and J), likely mediated by either attenuating the inflammatory changes that lead to an increase in LCN2 or reducing the deposition of tau pathology that triggers this response, or a combination of both. Together, our data show that neuronal health and aggregation of disease-related proteins can be modulated in a non–cell-autonomous fashion by astrocyte-secreted HSPB1. Our findings highlight a unique route by which astrocytes secrete HSPB1 to the medium with autocrine and paracrine protective functions (Fig. 8K).

## DISCUSSION

Astrocytes contribute to neuronal function in health and disease conditions through non–cell-autonomous mechanisms. Growing evidence supports a role of chaperones in mediating such effects, as shown in mouse and fly models of Huntington’s disease (*56*–*58*) or in SOD1 cellular models of amyotrophic lateral sclerosis (ALS) (*59*). Our work provides insight into this astrocyte-neuron axis and suggests that in diseases such as in AD, astrocytes have developed a strategy to counteract their own inflammatory response, maintain neuronal health, and prevent the aggregation of disease proteins in neurons such as tau through a mechanism that relies on the secretion of HSPB1 to the medium. While reactive astrocytes may have deleterious roles in AD, some of their responses, including secretion of HSPB1, may serve as an initial attempt to neutralize disease pathology.

HSPB1 is intimately linked to neurodegeneration. Mutations in HSPB1 are reported in motor neuron diseases, including distal hereditary motor neuropathy type II (dHMN) and the axonal form of Charcot-Marie Tooth disease (CMT2), as well as in ALS (*60*). Proteomic profiling of 14,513 proteins identified HSPB1 as one of the 58 differentially expressed proteins in human AD brain (*34*), and machine learning approaches have suggested HSPB1 as one of the two biomarkers to discriminate AD, together with amyloid precursor protein (APP) (*61*). When expressed under the cytomegalovirus (CMV) ubiquitous promoter, HSPB1 reduced the plaque load and rescued the long-term potentiation and spatial learning impairment in an APP/PS1 mouse model (*19*), an effect that may be mediated by its ability to bind Aβ in vitro and prevent its toxicity (*25*). HSPB1 has been reported to be secreted in exosomes from primary astrocytes in response to Aβ (*42*), and in cultured cells, it can modulate APP processing and secretion (*62*).

Our data show that, in AD brain, HSPB1 is predominantly expressed in astrocytes, in line with previous immunohistochemistry studies in AD and other tauopathies, where HSPB1 expression was mostly attributed to glial cells (*24**,* *63*), and with recent single-cell transcriptomic studies in human AD brain (*64**–**66*). In this work, we shed light on the functional implications of HSPB1 expression in astrocytes and its relationship with the local environment around amyloid plaques, rich in Aβ oligomers, dystrophic neurites, reactive astrocytes, and activated microglia (*23*). HSPB1 is expressed in GFAP astrocytes, and these numbers increased in the proximity of amyloid plaques. Increased levels of HSPB1 in astrocytes are a common feature in other tauopathies such as progressive supranuclear palsy or corticobasal degeneration (*67**–**69*). Astrocytic HSPB1 also increases when tau pathology in mice is restricted to neurons (*70*), suggesting that astrocytes respond with changes in HSPB1 across brain disease pathology and in response to a variety of stimuli.

Our data in human postmortem tissue, primary rodent cultures, and human cells from iPSC provide strong evidence for the secretion of HSPB1 from astrocytes. While we cannot discard that endothelial cells may also contribute to the extracellular HSPB1 found in human brain, data extracted from the Atlas Brain Map show that changes in gene expression across disease progression are mainly observed in astrocytes, suggesting that astrocytes may be the main source for any increase in extracellular HSPB1 in AD. We have previously shown that astrocytes increase the secretion of proteins with chaperone activity in response to Aβ (*71*). Astrocytes become reactive in response to a variety of extrinsic signals (*2*), including the presence of Aβ oligomers or an inflammatory environment. We propose that conditions leading to astrocyte reactivity also result in increased secretion of chaperones such as HSPB1. Given that HSPB1 increase has been reported in astrocytes across multiple neurological diseases with astrocyte reactivity, including AD (*24**,* *63*) and other tauopathies (*67**–**69*), Parkinson’s disease (*72*), or in brain ischemia (*73*), we can speculate that this is a conserved mechanism across diseases. Previous evidence supports the idea of HSPB1 as an extracellular protein with disease relevance: Elevated levels of HSPB1 are found in plasma after severe trauma (*74*), chronic obstructive pulmonary disease (*75*), acute ischemic stroke (*76*), or during attacks in multiple sclerosis (*77*), as well as in various types of cancer including pancreatic carcinoma (*78*) or gastric adenocarcinoma (*79*). Our literature review of the CSF proteome also points to HSPB1 as a common component of the brain extracellular space. While it is plausible that a fraction of HSPB1 is secreted within EVs as previously documented for different cell types (*42*–*44*), or through alternative secretion mechanisms that could be mediated by its interaction with the plasma membrane (*80*), our data suggest that most astrocytic HSPB1 is not secreted within vesicles. Instead, its secretion may be mediated through a mechanism that requires the lysosomal machinery as previously suggested for HSPB1 secretion from macrophages or endothelial cells in response to stimuli such as estrogens (*48*, *49*). While we do not rule out a direct function of secreted HSPB1 by acting on proteins present in the extracellular space, as it has been previously suggested (*25**,* *42**,* *62*), here, we have studied its effect in neighboring cells. Our data show that HSPB1 is taken up by both neurons and astrocytes. In human brain, HSPB1 is only found in a small proportion of neurons, suggesting that the contribution of internalized HSPB1 to overall protein levels inside these cells may be small, potentially because it is quickly degraded in the endolysosomal compartment.

In line with previous literature showing that HSPB1 could modulate the inflammatory response in different cell types (*18*), our data suggest that extracellular HSPB1 ameliorates some of the reactive astrocyte signature including a reduction in levels of proteins typically increased in reactive astrocytes such as LCN2 or Serpina3N and decreased secretion of markers of an inflammatory response such as NO and cytokines. Moreover, while the inhibitory effect of HSPB1 on tau fibril formation and aggregation has been reported in vitro and in cells (*7*–*9*), we propose an effect of extracellular HSPB1 on tau pathological inclusions in neurons. Our data suggest that HSPB1 can participate in a form of transcellular signaling to maintain neuronal proteostasis. A key question emerging from our data is the downstream effect of astrocyte-secreted HSPB1 and its potential mechanisms. An internalization of HSPB1 by astrocytes and neurons and its localization in acidic compartments would be compatible with its binding to membrane receptors to initiate downstream signaling pathways that could be followed by receptor-mediated endocytosis. It has been suggested that HSPB1 modulates the inflammatory response through binding to Toll-like receptors (TLRs) in vascular endothelial cells (*81**,* *82*) and dendritic cells (*83*) or through scavenger receptor A in macrophages (*48*). Binding of extracellular HSPB1 to receptors in the astrocyte surface such as TLRs may explain its impact on cytokine secretion, but it could also explain some of the effects observed in neurons, where TLR activation can modulate neurite outgrowth (*84*). Alternatively, the observed effects may be mediated by HSPB1 reaching the cytoplasm, for example, by lysosomal rupture in response to tau pathology, and having a direct function. In support of this, our data show that extracellular HSPB1 shares some functions with those reported for the intracellular HSPB1. HSPB1 has been reported to modulate pathways that control the inflammatory response (*18*), neurite outgrowth through binding to cytoskeleton components (*16**,* *85*), and binding to tau to prevent its aggregation (*7*–*9*). This suggests that extracellular HSPB1 can reach the intracellular compartment in neurons, similar to what happens to other extracellular chaperones such as clusterin (*86*) and proteins including tau or α-synuclein, which have been suggested to induce vesicle rupture following endocytosis (*87**,* *88*).

Treatment with recombinant HSPB1 has shown translational potential, reducing the paralytic symptoms when injected intraperitoneally in the experimental autoimmune encephalomyelitis mouse model, alongside decreased levels of the inflammatory cytokines IL-2, IL-6, and IFNγ (*89*). Intravenous infusion of human-derived HSPB1 or recombinant HSPB1 with a cell-penetrating peptide reduced blood-brain barrier dysfunction and improved neurological deficits, including reduced glia activation in mice following brain ischemia (*90**,* *91*). Our findings provide proof of concept for HSPB1 as a therapeutic target in AD and other tauopathies. Increasing the levels of HSPB1 in the brain extracellular space through strategies to deliver recombinant protein or using astrocyte-directed gene therapy vectors constitutes potential therapeutic avenues in AD and related tauopathies.

## MATERIALS AND METHODS

### Postmortem human brain

Paraffin-embedded postmortem human sections from the temporal cortex of age-, gender-, and postmortem delay–matched confirmed Braak 0-VI cases (table S1) were obtained from the London Neurodegenerative Diseases Brain Bank at King’s College London. All tissue donations were collected with informed consent for use in research, and this study was conducted under the ethical approval of the tissue bank (23/WA/0124 Wales REC 3).

### Systematic search of human CSF proteomic studies

Publicly available proteomics data were collected using PubMed searching for “(cerebrospinal fluid OR CSF) AND (proteome OR proteomics) AND (human) AND (normal OR healthy OR control),” with the filters “Full Text,” “Humans,” and “English.” This search yielded a total of 687 articles. Three additional papers were included, which had been cited in other studies, resulting in a total of 690 papers. Studies that were not proteomics analysis of total CSF (*n* = 390), did not involve human CSF (*n* = 18), did not include samples from healthy individuals or health status was not reported or was unclear (*n* = 41), or identified fewer than 1000 proteins (*n* = 226) were excluded, resulting in a final list of 15 studies, of which 2 used the same dataset.

### Estimation of sHSP cellular gene expression from single-cell RNA sequencing studies in human brain

Gene expression in single cells was retrieved from publicly available single-cell RNA sequencing data at the Cell Types Database from the Allen Brain Map portal (https://portal.brain-map.org/): human Multiple Cortical Areas SMART-seq (2019), human Primary Motor Cortex 10x Genomics (2020), and human Middle Temporal Gyrus references from the Seattle Alzheimer’s Disease Brain Cell Atlas (SEA-AD) (2022) (*92**–**94*). Trimmed means were extracted for all members of the sHSP family for the different cell populations, based on Cell Type Taxonomies in Allen Brain MAP (https://knowledge.brain-map.org/celltypes): neuronal (GABAergic and glutamatergic neurons), nonneuronal (astrocytes, oligodendrocyte precursor cells, and oligodendrocytes), and nonneural (microglia/perivascular macrophages, endothelial cells, and vascular and leptomeningeal cells). To determine gene expression changes in relation to AD progression, we have obtained the beta coefficient for either astrocytes or endothelial cells for all early and late disease pseudoprogression, available at the AD Gene Expression Trajectory Viewer (https://sea-ad.shinyapps.io/ad_gene_trajectories/), from the SEA-AD project (*94*).

### Animals

All animal work was conducted in accordance with the UK Animals (Scientific Procedures) Act 1986 Amendment Regulations 2021 under UK Home Office Personal and Project Licenses and approved by the King’s College London Animal Welfare and Ethical Review Board.

### Primary mouse cultures and treatments

#### Mouse primary glial cultures

Primary mixed glial cultures were prepared from the cortex of a pool of wild-type CD1 mice on postnatal days 1 to 3, as previously described (*71*). Briefly, brains were harvested and placed in ice-cold Dulbecco’s modified Eagle’s medium (DMEM), meninges were removed, and cerebral cortices were dissected, pooled together, and mechanically dissociated. Glial cells were grown in DMEM (Gibco, 21969-035) supplemented with 2 mM GlutaMAX (Gibco, 35050061), penicillin (100 U/ml), streptomycin (100 μg/ml; Gibco, 15140122), and 10% fetal bovine serum (FBS) (Gibco, 10500064), in poly-d-lysine (PDL; 50 μg/ml; Sigma-Aldrich, P6407)–treated flasks. To prepare astrocyte cultures, 14 to 28 DIV mixed glial cultures were shaken at 220 rpm overnight to remove microglia. Astrocyte-enriched cultures (>95% GFAP-positive) were trypsinized in TrypLE (Gibco, 12605010) and seeded in PDL-coated plates for 3 days until they reached 80 to 90% confluency. To prepare primary microglia cultures, 7-DIV mixed glial cells were treated with granulocyte-macrophage CSF (GM-CSF; 5 ng/ml; PeproTech, 315-03-50) to enhance microglia proliferation, and medium was replaced with fresh GM-CSF after 4 days. After 3 days, flasks were shaken at 220 rpm for 3 hours, and microglia cells were grown in PDL-coated plates. All cultures were maintained at 37°C in a humidified incubator with 5% CO_2_. For treatments, astrocyte and microglia culture medium was replaced by complete DMEM containing 1% FBS.

Astrocytes were treated with TNFα (30 ng/ml, R&D Systems, 410-MT-050) and/or IL-1α (3 ng/ml, Sigma-Aldrich, I3901), Baf-A1 (20 nM in DMSO, Alfa Aesar, J61835.M), rhHSPB1 [50 ng/ml in 0.1% (w/v) bovine serum albumin (BSA) in phosphate-buffered saline (PBS), R&D Systems, 1580-HS], or heat-inactivated rhHSPB1 prepared by incubating at 99°C for 30 min. For live-cell experiments, rhHSPB1–Oregon Green (120 ng/ml) was used. Microglia cultures were treated with LPS (0.1 μg/ml, Sigma-Aldrich, L5293) and IFNγ (0.01 μg/ml, eBioscience, 14-8311) for 6 hours, and cells were then washed to eliminate any remaining LPS and IFNγ and incubated for further 18 hours in complete DMEM with 1% FBS.

#### Mouse primary neuron cultures

Primary neurons were prepared from the cortex of wild-type CD1 embryos on E16.5 as previously described (*71*). Briefly, brains were harvested and placed in ice-cold Hanks’ balanced salt solution with Hepes, where the meninges were removed, and cerebral cortices were dissected and digested in TryplE (Gibco, 12605010). Cells were cultured in poly-d-lysine (Sigma-Aldrich, P7280)–coated plates and grown in Neurobasal medium (Gibco, 21103049) supplemented with 2% B27 (Gibco, 17504044), GlutaMAX (Gibco, 35050061), sodium pyruvate (Gibco, 11360070), penicillin (100 U/ml), and streptomycin (100 μg/ml; Gibco, 15140122) at 37°C in a humidified incubator with 5% CO_2_ until 12 to 14 DIV. For treatment with astrocyte-conditioned medium, 40% of neuronal medium was replaced with medium from astrocytes grown in Neurobasal medium. For neurite growth assays, 0.1% BSA or rhHSPB1 (10 ng/ml) was directly added to neuronal medium at 3, 6, 9, and 12 DIV and fixed at 14 DIV. Neurons at 6 DIV were transduced with AAVs to express wild-type (AAV2/TM8-WT-htau0N4R-EGFP) or mutant (AAV2/TM8-P301L/S320F-htau0N4R-EGFP) htau at 2.5 × 10^10^ viral genome (VG)s/ml, followed by treatment with either rhHSPB1 (50 ng/ml in 0.1% BSA, R&D Systems, 1580-HS) or vehicle (0.1% BSA in PBS), at 6 DIV and every 48 hours until 12 DIV.

### DNA and siRNA transfection

Primary mouse astrocytes were transfected with pCMV.FRT-V5-hHSPB1 (a gift from H. Kampinga, Addgene plasmid no. 63102) or an empty vector (pcDNA3.1) for 24 hours in DMEM with 10% FBS, followed by further 24 hours in DMEM with 1% FBS or complete Neurobasal medium with 2% B27. Astrocytes were transfected with 50 nM On-Target Plus SMART pool (Dharmacon) siRNAs for mouse *HSPB1* (L-045651-00) or nontargeting (Dharmacon, D-001810-10) for 72 hours in complete DMEM with 10% FBS, followed by further 24 hours in DMEM with 1% FBS.

### Labeling of rhHSPB1 with Oregon Green and live-cell imaging

rhHSPB1 (R&D Systems, 1580-HS) manufacturer stock solution was exchanged to 0.1 M sodium bicarbonate (pH 8.3) using a 3-kDa cutoff filter (Millipore, UFC500396) and incubated in Oregon Green 488 dye (1 mg/ml; Thermo Fisher Scientific, O6149) for 1 hour at room temperature, and unbound Oregon Green was washed in PBS using a 3-kDa filter column until clear. Cells in 96-well plates were treated with rhHSPB1–Oregon Green together with 100 nM LysoTracker Red DND-99 (Thermo Fisher Scientific, L7528) and immediately imaged with an Opera Phenix High-Content Screening System (Perkin-Elmer) with environment maintained at 37°C with humidified CO_2_.

### iPSC culture and differentiation into astrocytes

#### LCL culture and iPSC reprogramming

Two control human lymphoblastoid cell lines (LCLs) were obtained from the European Collection of Authenticated Cell Cultures. Cells were donated by two healthy males that were over the age of 40 at the time of collection (cell lines: C53 and C70). LCLs were cultured in nonadherent T25 tissue culture flasks at 37°C and 5% CO_2_ in LCL medium (RPMI 1640, Gibco, 21875034), supplemented with GlutaMAX (Gibco, 35050061) and 20% FBS (Gibco, 10500064). LCLs were reprogrammed into iPSCs, as previously reported (*95*). Briefly, LCLs were transfected with plasmids expressing *OCT3/4*, *L-MYC*, *KLF4*, *SV40LT*, *LIN28*, *SOX2*, and *shRNA-p53* [namely, pEP4 E02S ET2K—a gift from J. Thomson (Addgene plasmid no. 20927), pCXLE-hOCT3/4-shp53-F, pCXLE-hUL, and pCXLE-hSK—a gift from S. Yamanaka (Addgene plasmid nos. 27077, 27080, and 27078] using the Amaxa Human B Cell Nucleofector Kit (Lonza, VPA-1001). Cells were transferred onto a feeder layer of mitomycin C–inactivated mouse embryonic fibroblast in LCL medium for 5 days. Medium was transitioned to reprograming medium as previously described. Approximately 2 weeks after electroporation, small colonies were detected, and the medium was transitioned to Essential 8 Flex medium (Gibco, A2858501). Colonies were manually picked and transferred to a plate coated with Geltrex (Thermo Fisher Scientific, A1413302) in Essential 8 Flex. iPSCs were routinely screened for mycoplasma contamination using the MycoAlert Mycoplasma Detection Kit (Lonza, LT07).

#### iPSC characterization

iPSCs displayed normal morphology and, as evidence of pluripotency, were shown to differentiate into cells from the three germ layers of the blastocyst, as previously described (*95*). Approximately 14 days after spontaneous differentiation, the cells were fixed and stained for endodermal cells expressing α-fetoprotein (1:200, Santa Cruz Biotechnology, sc-8108), mesodermal cells expressing smooth muscle actin (1:200, Abcam, ab5694), and ectodermal cells expressing β3-tubulin (1:400, Sigma-Aldrich, T8660). iPSCs were subjected to digital karyotyping using the KaryoStat+ Karyotyping Service (Thermo Fisher Scientific, A52849), which did not indicate any chromosomal abnormalities.

#### Astrocyte differentiation from iPSCs

iPSCs were cultured in Essential 8 Flex medium and differentiated into astrocyte progenitors, as described by Serio *et al.* (*41*), with a few modifications. iPSCs were initially differentiated into neural precursors (NPCs) until astroglial progenitors (APCs) were observed. APCs are larger than neurons and adhere better to the plate. Hence, APCs were purified by passaging the mixed population of NPCs and APCs at low density, leading to an enrichment of APCs over NPCs. Cells were cultured and passaged at low density for 2 to 4 weeks to obtain a pure population of APCs. APCs were seeded onto Matrigel (Corning, 356234)–coated plates and terminally differentiated into astrocytes by the addition of CNTF (10 ng/ml; PeproTech, 450-13) for at least 2 weeks. Astrocyte progenitors were positive for vimentin (Sigma-Aldrich), and mature astrocytes were positive for GFAP (Abcam, ab4674). Medium from mature astrocytes was replaced by fresh medium and collected after 24 hours.

### Organotypic brain slice culture and treatments

Organotypic brain slice cultures were prepared from wild-type CD1 mice on P7, as previously described (*53*, *55*). The sex of the pups was not determined. Brains were dissected in ice-cold oxygenated dissection buffer [124 mM NaCl, 3 mM KCl, 1.25 mM KH_2_PO_4_, 8.2 mM MgSO_4_·7H_2_O, 2.65 mM CaCl_2_·2H_2_O, 3.5 mM NaHCO_3_, 1.99 mM ascorbic acid, 10 mM glucose, ATP (0.2 mg/ml; Sigma-Aldrich, A6419); pH 7.4], and three 350-μm-thick sections cut with a Mcllwain tissue chopper (Stoelting, 51350) were positioned on a 0.4-μm Millicell culture insert (Merck Millipore, 10412511) and cultured in 1 ml of slice culture medium [19.3 mM NaCl, 5 mM NaHCO_3_, 2.7 mM CaCl_2_·2H_2_O, 2.5 mM MgSO_4_·7H_2_O, 0.5 mM ascorbic acid, basal medium Eagle (9.19 g/liter; Sigma-Aldrich, B9638), 40 mM glucose, 1 mM Hepes (Sigma-Aldrich, H0887), penicillin-streptomycin (5 ml/liter; Sigma-Aldrich, 15140122), insulin (0.33 ml/liter; Sigma-Aldrich, 9278), and 25% horse serum (Sigma-Aldrich, H1138); pH 7]. Three to four hippocampal slices were separated from the cortex in dorsal slices. The medium was replaced after 2.5 hours. AAV8-GFAP-BFP (1 × 10^10^ VGs/ml) and AAV8-GFAP-BFP:hHSPB1 (1 × 10^10^ VGs/ml) or AAV2/8-WT-EGFP (1 × 10^11^ VGs/ml) and AAV2/8-P301L/S320F-htau-EGFP (1 × 10^11^ VGs/ml) were added at 0 DIV, and the medium was replaced every 2 to 3 days until 21 DIV for hHSPB1 AAVs and 28 DIV for htau AAVs. After 14 DIV, to allow for any stress caused by the axotomy to be resolved (*53*), slices were treated with rhHSPB1 (50 ng/ml) or 0.1% BSA and at each medium change (until harvesting at 28 DIV), or treated with either TNFα + IL-1α or rhHSPB1 for 24 hours in complete Neurobasal medium with 2% B27 and collected at 21 DIV. Treatments and controls were region-matched with slices from each hemisphere.

### Recombinant AAVs

AAV8-GFAP-BFP and AAV8-GFAP-BFP:hHSPB1 were obtained from VectorBuilder by packaging pAAV[Exp]-GFAP(short)>TagBFP2: WPRE (VectorBuilder, VB210218-1066qmw) and pAAV[Exp]-GFAP(short)-hHSPB1[NM_001540.5]:P2A:TagBFP2:WPRE (VectorBuilder, VB210211-1047trt) into single-stranded AAV8 viruses. AAV2/8-WT-EGFP and AAV2/8-P301L/S320F-htau-EGFP to express the 0N4R htau under the hCBA promoter were obtained by microscale preparation (*54*).

### Immunohistochemistry of human brain sections

Temporal cortex sections (7 μm) of human AD and control brain were prepared from formalin-fixed paraffin-embedded blocks. Sections were deparaffinized in xylene (Thermo Fisher Scientific, X/0250/17) and rehydrated in 99% (v/v) ethanol, followed by antigen retrieval in citrate buffer (0.01 M sodium citrate, pH 6) at 95° to 100°C for 5 min and 65°C for 12 min. Sections were cooled in water and rinsed in tris-buffered saline (TBS), and a hydrophobic wax boarder was applied using PAP pen (SLS, HIS0500). Sections were placed in blocking buffer [1:100 normal goat serum (Sigma-Aldrich, G9023) in TBS] followed by primary antibody incubation overnight: 6E10 (1:200, BioLegend 803002), ALDH1L1 (1:200, Antibodies-online ABIN1304519), CAII (1:100, Abcam ab124687), GFAP (1:500, Dako N1506 or Abcam ab4674), HSPB1 (1:200 Enzo ADI-SPA-803 or HSPB1 mouse Proteintech, 66767-1-Ig), IBA1 (1:200, Wako 019-19741), and MAP2 (1:500, Sigma-Aldrich 05-346 or GeneTex GTX82661). After fluorophore-coupled secondary antibody (Invitrogen) incubation at 1:250, sections were treated with 0.3% (w/v) Sudan Black (Acros Organics, 190160250) in 70% ethanol and mounted using Fluoromount-G mounting medium with 4′,6-diamidino-2-phenylindole (DAPI) (Invitrogen, 00-4959-52).

### Analysis of human brain sections

Images were taken with either a Zeiss Axioscope-Apotome microscope or a Nikon Ti-E camera inverted epifluorescence microscope with a 40× oil immersion objective. For quantification, images were taken in a Nikon Eclipse Ti inverted spinning disk confocal microscope with 40× oil immersion/1.4–numerical aperture (NA) objective and Z-stacks, including regions with an amyloid plaque in the center and 50 μm from the plaque edge, referred to as proximal to plaques. Distal regions were selected from equivalent adjacent areas, where no plaques are found within 50 μm. A minimum of 10 plaques and equivalent control areas were taken from each case. Quantification of mean fluorescence intensity was performed in ImageJ. For the analysis of extracellular HSPB1, Z-stacks were projected with maximum intensity. Nuclear and GFAP staining were used to manually distinguish single astrocytes, and a 50-μm diameter was defined as the surrounding area. A mask was created using GFAP, and the mean intensity of HSPB1 signal outside the mask was quantified in the selected area in approximately 50 astrocytes per case and condition.

### Collection of conditioned medium, cultured cells, and brain slice lysates

The medium from astrocytes, microglia, or brain slices was centrifuged at 1000*g*, 4°C for 5 min to eliminate any dead cells and debris. The supernatant was stored at −20°C. For Western blotting, the medium was concentrated approximately 10 times in a 30-kDa molecular weight cutoff column (Millipore, UFC503096) and resuspended in Laemmli buffer. Cell lysates were collected in Laemmli buffer. Three brain slices in one insert were collected in radioimmunoprecipitation assay buffer (Thermo Fisher Scientific, 89900) supplemented with protease inhibitors (Roche, 11836170001) and phosphatase inhibitors (Roche, 4906845001) and homogenized in a polytetrafluoroethylene tissue grinder (Fisherbrand, 10075911). Protein concentration was measured using a bicinchoninic acid assay (Thermo Fisher Scientific, 23227) and resuspended in Laemmli buffer.

### Western blotting

Protein extracts were separated by 10 or 12% SDS–polyacrylamide gel electrophoresis in a Mini-PROTEAN Tetra system (Bio-Rad) and transferred to 0.45-μm nitrocellulose membrane (Amersham Protran, 10600002) in a Mini Trans-Blot system (Bio-Rad). After blocking in Odyssey blocking buffer (LI-COR, 927-60003), blots were probed with primary antibodies 1:1000: V5 (Invitrogen, R960-25), mouse HSPB1 (Enzo, ADI-SPA-801), human HSPB1 (Enzo, ADI-SPA-803), phospho-HSPB1 (S82) (D1H2F6) (CST, 9709), LCN2 (R&D Systems, AF1857), SerpinA3N (R&D Systems, AF4709), iNOS (Abcam, ab3523), glyceraldehyde-3-phosphate dehydrogenase (GAPDH; Santa Cruz Biotechnology, sc-32233), β-actin (Abcam, ab8227 and ab8226), ALDH1L1 (Antibodies-online, ABIN1304519), IBA1 (Wako, 019-19741), GFAP (Dako, Z0334), β-III-tubulin (Abcam, ab18207), CD81 (Santa Cruz Biotechnology, sc-166029), tau (Dako, A0024), and PHF-1 (P. Davies). Blots were washed in TBS with 0.1% Tween 20 and incubated with the appropriate IRDye 800–or IRDye 680–conjugated anti-mouse, anti-rabbit, or anti-goat secondary antibodies (LI-COR) diluted 1:10,000. Signal was visualized using an Odyssey CLx infrared imager (LI-COR) and quantified using ImageStudio Lite (Li-COR) software.

### Immunofluorescence of cultured cells

Cells were fixed with 4% (w/v) paraformaldehyde (PFA; Avantor, 43368.9M) and permeabilized with 0.1% (v/v) Triton X-100 in PBS followed by blocking in 5% (w/v) BSA (Sigma-Aldrich, A7906) and incubation with primary antibodies: NF-κB p65 (1:500, Cell Signaling Technology, 6956), human HSPB1 (1:500, Enzo, ADI-SPA-803), MAP2 (1:1000, GeneTex GTX82661), and MC1 (1:500, P. Davies). Secondary antibodies (Invitrogen) were applied at 1:1000 followed by Hoechst (0.1 μg/ml, Invitrogen, 33342) and mounted with fluorescence mounting medium (DAKO S3203). For LC3 and LAMP1, the cells were fixed with cold methanol at 4°C, followed by blocking in 10% (v/v) goat serum: LAMP1 (1:500, Developmental Studies Hybridoma Bank, 1D4B), LC3 (1:500, Nanotools, 0231-100/LC3-5F10), and mouse HSPB1 (1:500, Enzo, ADI-SPA-801). For NF-κB p65, samples were imaged using Zeiss Axioscope-Apotome microscope. For MAP2 neurite outgrowth, 96-well plates were imaged using an Opera Phenix High-Content Screening System (PerkinElmer) using 20× water objective. LC3, Lamp1, and HSPB1 staining were imaged using Nikon Inverted A1R confocal microscope with 63× oil immersion/1.4-NA objective and Z-stack images.

### Immunofluorescence of mouse organotypic brain slice cultures

Brain slices were fixed in precooled 4% PFA inside and outside the insert at room temperature for 20 min, followed by incubation in precooled 20% methanol overnight at 4°C. Insert membrane was cut, and slices were permeabilized in 0.5% (v/v) Triton X-100 in PBS overnight at 4°C, washed, and blocked in 20% (w/v) BSA in PBS supplemented with 0.1% Triton X-100 overnight at 4°C. Brain slices were then incubated for 72 hours at 4°C with 1:200 primary antibodies diluted in 5% BSA in PBS supplemented with 0.1% Triton X-100: hHSPB1 (Enzo, ADI-SPA-803) and GFAP (Abcam, ab4674). Slices were washed with 5% BSA in PBS and incubated with 1:500 secondary antibodies (Invitrogen) at room temperature for 4 hours. After washing in 5% BSA in PBS, the slices were incubated with SYTO Deep Red (Invitrogen, S34900) (1:200 in 5% BSA in PBS) at room temperature for 30 min. Brain slices were mounted onto glass slides with ProLong Gold Antifade Mountant (Thermo Fisher Scientific, P36930). Samples were imaged using Nikon Inverted A1R confocal microscope with 63× oil immersion/1.4-NA objective and Z-stack images.

### RNA isolation and quantitative PCR

RNA was extracted from primary mouse astrocytes using the Absolutely Total RNA Purification Kit (Agilent, no. 400800). cDNA was synthesized from 200 ng of extracted total RNA reactions using High-Capacity RNA-to-cDNA Kit (Thermo Fisher Scientific, no. 4388950). Quantitative polymerase chain reaction (PCR) was performed with the PowerUp SYBR Green Master Mix (Thermo Fisher Scientific, no. A25741) by QuantStudio 7 Flex Real-Time PCR System (Thermo Fisher Scientific, no. 4485701). GAPDH was used as an internal control gene. The relative fold change in mRNA between conditions was calculated and presented as 2^−ΔΔCt^. ΔΔCt = ΔCt (treatment) − ΔCt (control). Primer sequences were as follows: *HSPB1*, 5′-GAGGAGCTCACAGTGAAGAC-3′ (forward) and 5′-GCGTGTATTTCCGGGTGAAG-3′ (reverse); *GAPDH*, 5′-GACGGCCGCATCTTCTTGTG-3′ (forward) and 5′-CAAATGGCAGCCCTGGTGAC-3′ (reverse).

### LDH assay

Cytotoxicity was measured using CytoTox 96 Non-Radioactive Cytotoxicity Assay (Promega, G1780) by quantitatively measuring LDH according to the manufacturer’s protocol. Absorbance was measured at 490 nm on a CLARIOStar plate.

### Griess assay

Griess reagent kit (Invitrogen, G7921) was used to quantify nitrite levels in the medium as an indirect measure of NO levels, following the manufacturer’s instructions. Absorbance was read at 548 nm on a CLARIOStar plate reader.

### ELISA to detect IL-6 levels

A murine IL-6 enzyme-linked immunosorbent assay (ELISA) Development Kit (PreproTech, 900-TM50) was used following the manufacturer’s instructions in a 96-well ELISA plate (BioLegend, 423501). The plate was read at 450 nm with the wavelength correction set at 620 nm on a CLARIOStar plate reader.

### Proteinase K protection assay

Equal volumes of concentrated astrocyte-conditioned medium were incubated on ice with increasing concentrations of proteinase K (Thermo Fisher Scientific, no. EO0491) for 30 min. Digestion was stopped by protease inhibitor (Roche, no. 11836170001). Samples were resuspended in Laemmli buffer and denatured with 95°C followed by Western blotting.

### Detection of NAG secretion

The presence of the lysosomal NAG in the medium was detected using a NAG assay kit (Sigma-Aldrich, CS0780) according to the manufacturer’s instructions. Before the assay, astrocytes were cultured in phenol red–free complete medium DMEM–1% FBS (DMEM, Gibco 31053028). Absorbance was read at 405 nm on a CLARIOStar plate reader.

### Isolation of EVs by SEC

Astrocytes were cultured in DMEM supplemented with 1% of EV-free FBS by previously FBS centrifugation at 100,000*g* for 18 hours at 4°C in a Beckman Coulter Optima XPN-80 ultracentrifuge, aliquoted. Astrocyte medium was centrifuged at 300*g*, 10 min at 4°C and then at 2000*g*, 10 min at 4°C, followed by concentration using an ultracentrifugal filter column (MilliporeSigma, no. UFC900308). The medium was loaded into a SEC column (qEV10 / 35 nm, Izon, no. SP7), and eluted fractions were collected. Protein concentration was measured using NanoDrop spectrophotometer. For Western blotting, each fraction was concentrated through a 3-kDa cutoff column (MilliporeSigma, UFC500396) and resuspended in Laemmli buffer. For membrane rupture, before SEC, Triton X-100 was added to concentrated medium at 1% (v/v) concentration and the medium was vortexed every 10 min and kept on ice for 1 hour.

### Nanoparticle tracking analysis

EVs-rich fractions (F7 to F9) were pooled and measured by NTA (NanoSight LM10 system) with sCMOS camera. Data were collected and analyzed using the NanoSight LM10 3.2 software. The camera level was set to 14 (NTA 3.0 level), and the detection threshold was set to 3 to reveal small particles. Ambient temperature was recorded manually, ranging from 19.8° to 20.1°C. The sample was diluted 1:200 in sterile PBS to ensure that the particle concentration was between 1 × 10^6^ and 1 × 10^9^ particles/ml. Each sample was recorded five times for 30 s, and histograms were averaged.

### Mouse cytokine array

A Proteome Profiler Mouse Cytokine Array Kit (R&D Systems, ARY006) was used according to the manufacturer’s instructions. Captured cytokines were visualized with 1:2000 IRDye 800cw Streptavidin (LI-COR, 926-32230) and imaged with the Odyssey infrared imaging system (LI-COR, Biosciences). Cytokine abundance was calculated as signal intensity of each dot plot relative to the mean of three reference spots, and *z* score was calculated.

### Analysis of neurite outgrowth

Harmony high-content analysis software (PerkinElmer) was used to analyze the MAP2-positive neurite properties identified with the Find Neurites building block. Parameters were calculated per neuron and averaged to total number of neurons imaged per well. All experiments were performed in 96-well plates with a minimum of five wells imaged per condition and per biological replicate.

### Measurement of tau aggregation in neurons

Neurons were fixed in 4% (w/v) PFA at room temperature, and nuclei were stained with Hoechst. Using a Zeiss Axioscope-Apotome microscope, the proportion of EGFP-positive neurons that contained mutant tau aggregates was counted. Samples were blinded, and a minimum of 500 cells was counted per replicate. Each experiment was performed in three technical replicates.

### Sarkosyl-insoluble tau extraction

Sarkosyl-insoluble tau was isolated from a pool of 12 mouse brain slices. Brain slices were harvested in ice-cold homogenization buffer [50 mM TBS, 10% (w/v) sucrose (Sigma-Aldrich, S9378), 2 mM EGTA (Sigma-Aldrich, E4378)], supplemented with protease (Roche, 11873580001) and phosphatase inhibitors (Roche, 4906845001) at 100 mg/ml and mechanically dissociated using a Teflon glass homogenizer. The supernatant was collected following centrifugation at 4°C for 20 min at 13,000*g*, and an aliquot was retained as the low-speed supernatant. *N*-lauroylsarcosine sodium salt solution (Sarkosyl, Sigma-Aldrich, L7414) was added to the remaining supernatant to a final concentration of 1% and incubated at ambient temperature for 30 min before centrifugation at 100,000*g* for 1 hour at 21°C using an Optima MAX-XP ultracentrifuge (Beckman Coulter). The supernatant was collected as the high-speed supernatant containing sarkosyl-soluble tau. The pellet was washed with 1% sarkosyl in homogenization buffer and centrifuged at 100,000*g* for 15 min at 21°C. The pellet, containing sarkosyl-insoluble tau, was resuspended in Laemmli buffer.

### Statistical analysis

Statistical analysis was performed using GraphPad Prism 8. For quantification of Western blot data, relative protein abundance was calculated as the signal intensity relative to a loading control. Data were normalized to allow for comparison across blots by dividing each value by the sum of all data points on the same membrane. Similar normalization was done for IL-6 values obtained by ELISA. Statistical analysis was done using data from biological replicates or individual human brain cases, and the number of replicates is indicated in figure legends. For data with one independent variable, a normality-and-lognormality test was performed to confirm the Gaussian distribution of the data; to compare two different groups, two-tailed Student’s *t* test was used; to compare more than two groups, a one-way analysis of variance (ANOVA) with Tukey’s multiple comparison was used. For data with two independent variables, a two-way ANOVA with Tukey’s or Sidak’s multiple comparisons was used. The confidence interval was 95%. *P < 0.05, **P < 0.01, ***P < 0.001, and ****P < 0.0001.
