# Supplementary material for: Reactive astrocytes secrete the chaperone HSPB1 to mediate neuroprotection
Source: Sci Adv. 2024 Mar 20;10(12):eadk9884. doi: 10.1126/sciadv.adk9884 (PMC10954207; doi:10.1126/sciadv.adk9884)
Supplement: Supplementary file 1 — Figs. S1 to S15 Table S1 [file sciadv.adk9884_sm.pdf]

Science Advances
AAAS

Supplementary Materials for

Reactive astrocytes secrete the chaperone HSPB1 to mediate neuroprotection

Fangjia Yang *et al.*

Corresponding author: Maria Jimenez-Sanchez, maria.jimenez_sanchez@kcl.ac.uk

Sci. Adv. **10**, eadk9884 (2024)
DOI: 10.1126/sciadv.adk9884

This PDF file includes:

Figs. S1 to S15
Table S1

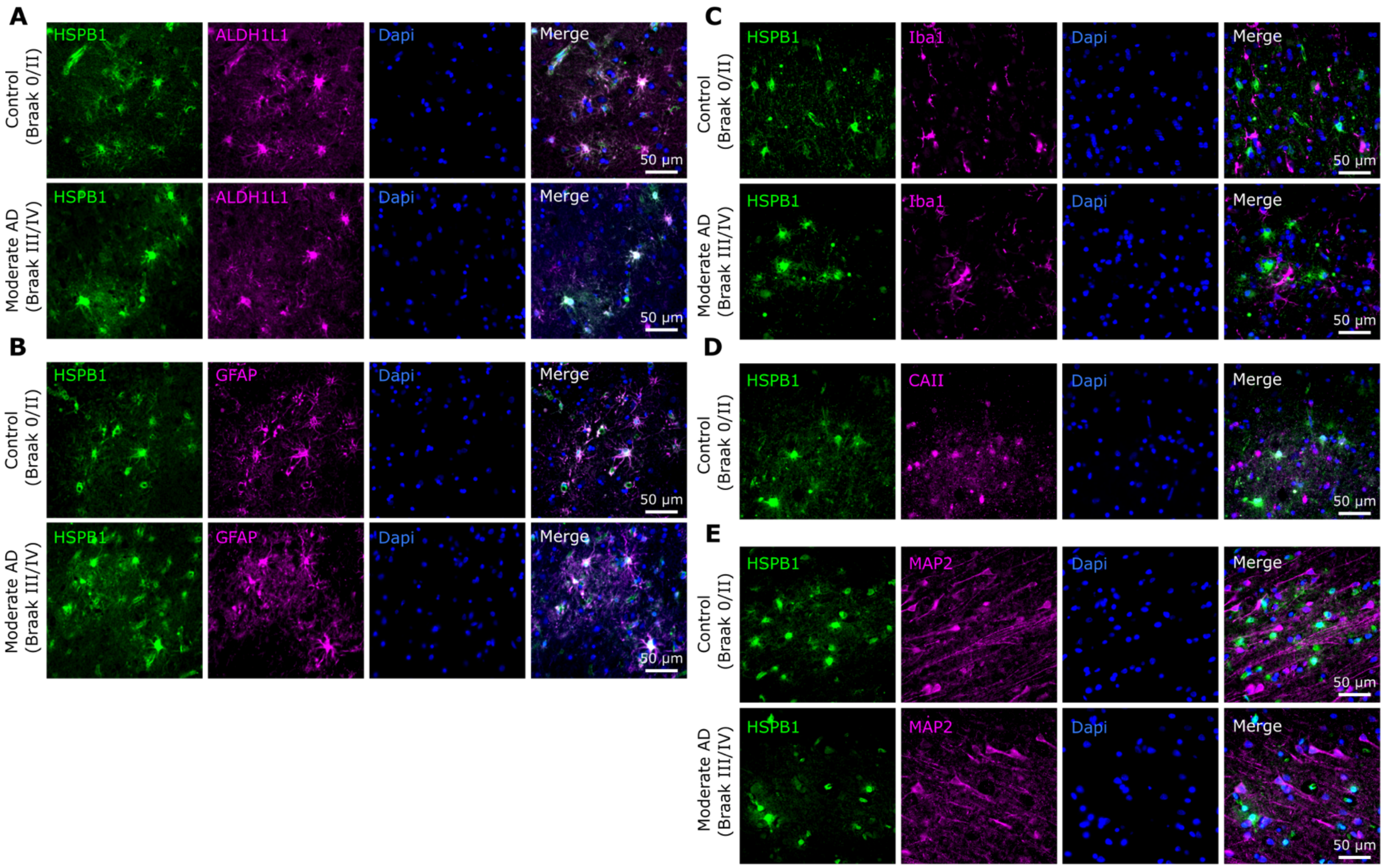

Figure S1. HSPB1 protein is predominantly detected in astrocytes in human brain in control and moderate cases of AD. Representative images of temporal cortex from control (Braak 0/II) or moderate AD (Braak III/IV) brain co-immunostained with HSPB1 and ALDHL1 (astrocytes) (**A**), GFAP (reactive astrocytes) (**B**), IBA1 (**C**), CAII (oligodendrocytes) (**D**) and MAP2 (neurons) (**E**), as indicated.

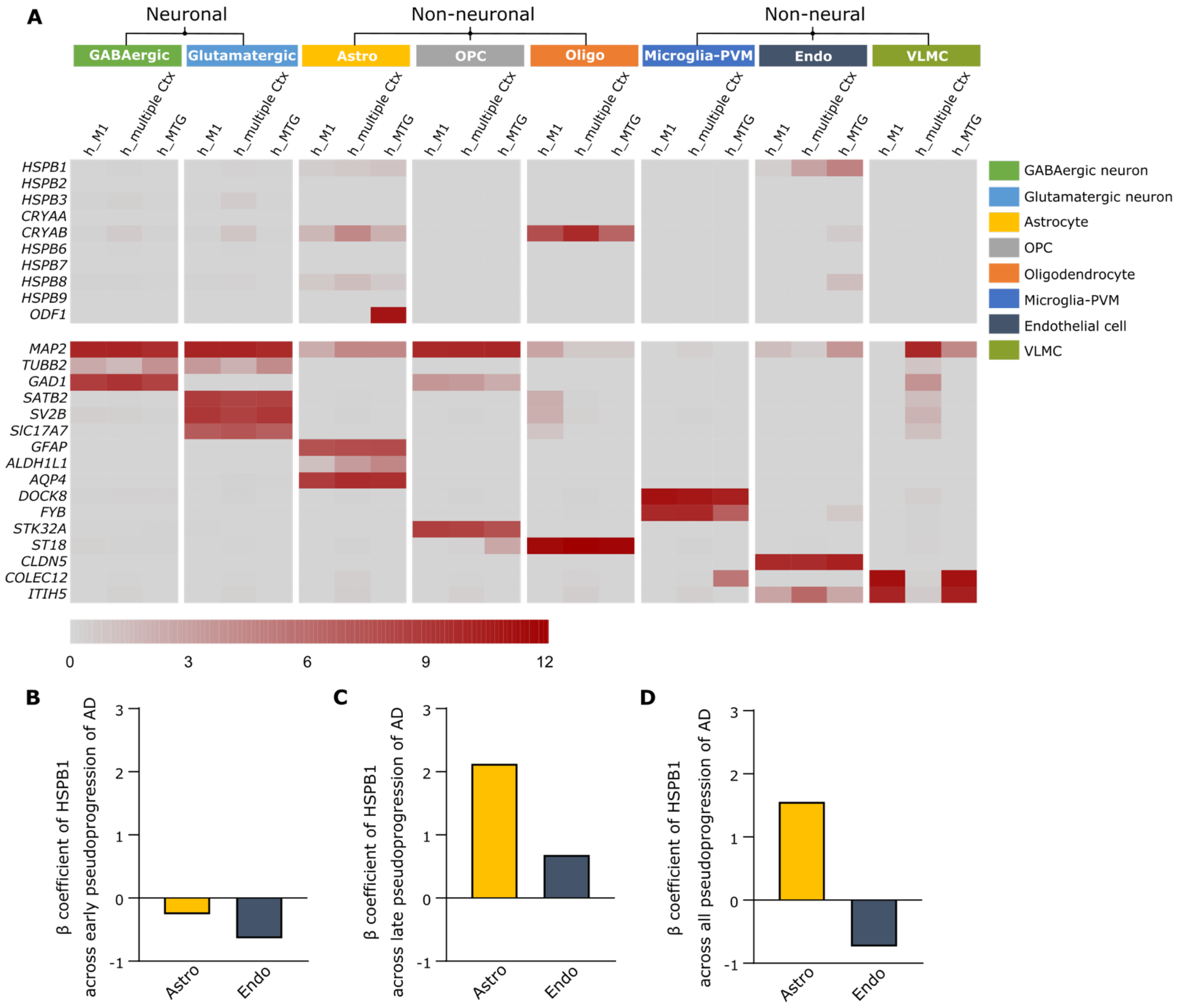

Figure S2. sHSPs gene expression data within brain cell subtypes shows HSPB1 in astrocytes and endothelial cells. A. For selected genes, expression in cell subtypes was retrieved from 3 publicly available scRNA-seq datasets from the Allen Brain Map portal: human Primary Motor Cortex (h_M1), human Multiple Cortical Areas (h_multiple Ctx) and human Middle Temporal Gyrus (MTG) (h_MTG). Trimmed mean expression for all members of the sHSP family is shown for the different cell populations: neuronal (GABAergic and glutamatergic neurons), non-neuronal (astrocytes, oligodendrocyte precursor cells (OPC), oligodendrocytes) and non-neural

(microglia/perivascular macrophages (PVM), endothelial cells and vascular and leptomeningeal cells (VLMC). Known marker genes for the different cell types is shown as a reference. **B-D**. Graphs show the β coefficient for early, late or all AD pseudoprogression for HSPB1 in astrocytes and endothelial cells, extracted from AD Gene Expression Trajectory Viewer (https://sea-ad.shinyapps.io/ad_gene_trajectories/). The β coefficient indicates how much a gene changes across early (**B**), late (**C**) or all (**D**) pseudoprogression in a given population. Positive values indicate increases in AD, while negative values indicate decreases.

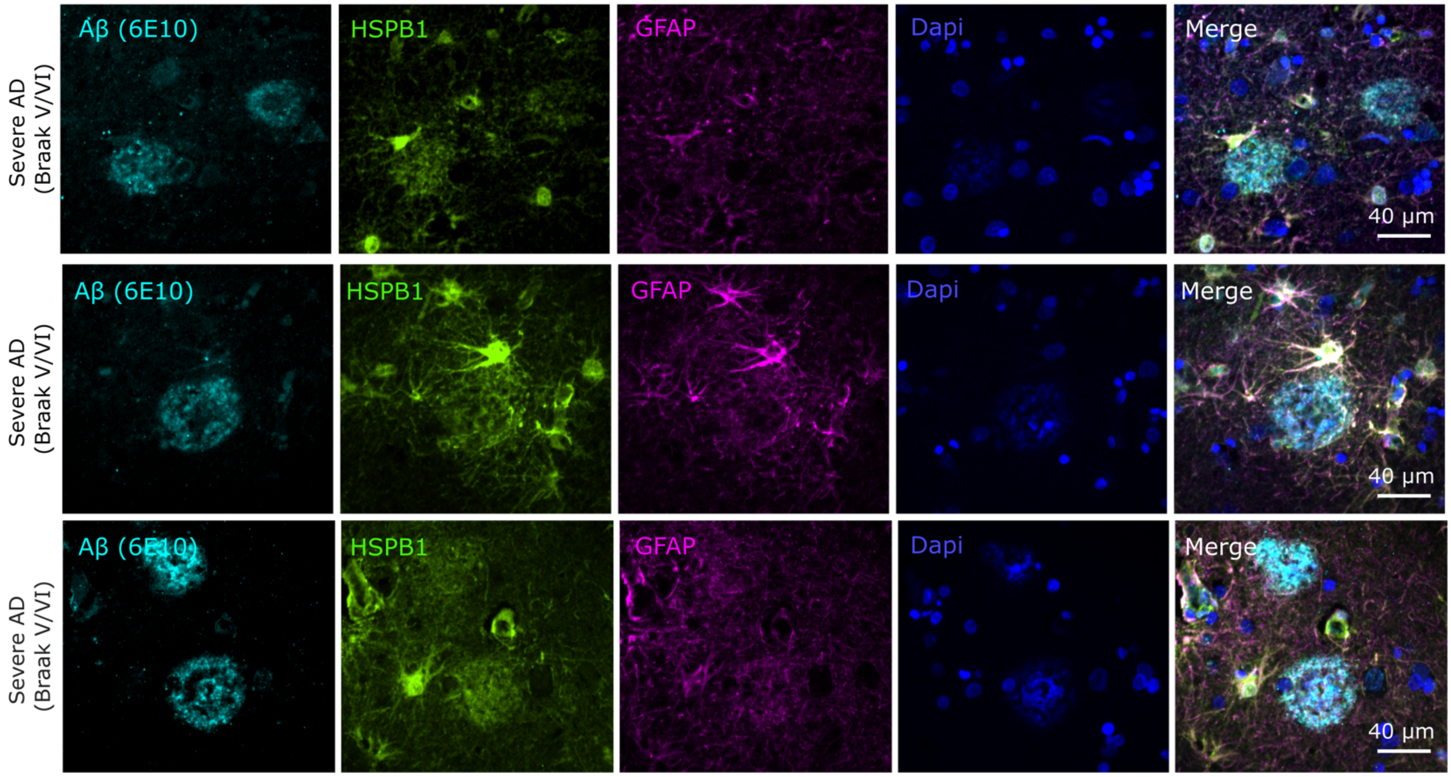

Figure S3. Amyloid β plaques in human AD brain are positive for HSPB1. Representative images of Braak V/VI AD cases where amyloid plaques are stained with a 6E10 antibody against Aβ and showing HSPB1 signal within amyloid plaques.

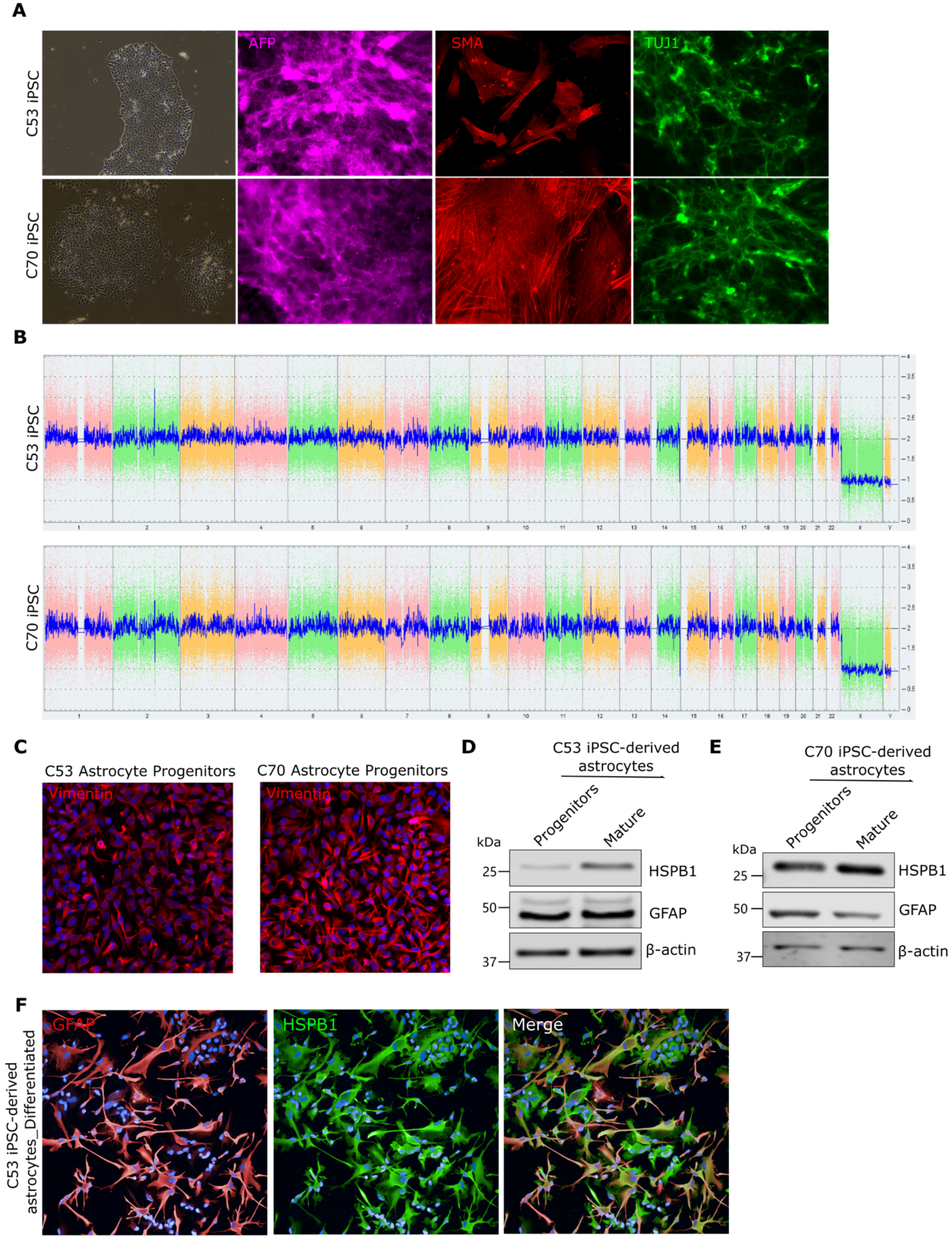
A
C53 iPSC
C70 iPSC
AFP
SMA
TUJ1
B
C53 iPSC
C70 iPSC
C
C53 Astrocyte Progenitors
C70 Astrocyte Progenitors
Vimentin
Vimentin
D
C53 iPSC-derived astrocytes
Progenitors
Mature
kDa
25
50
37
HSPB1
GFAP
β-actin
E
C70 iPSC-derived astrocytes
Progenitors
Mature
kDa
25
50
37
HSPB1
GFAP
β-actin
F
C53 iPSC-derived astrocytes_Differentiated
GFAP
HSPB1
Merge

Figure S4. iPSC characterisation and differentiation into mature astrocytes. A. Phase contrast images of C53 and C70 control iPSC lines, showing typical iPSC morphology with small round cells and large nuclei, growing in defined colonies. iPSCs were allowed to spontaneously differentiate and cultures were probed with immunofluorescence for alpha-fetoprotein (AFP, magenta), alpha-smooth muscle actin (SMA, red), and β-III-Tubulin (Tuj1, green), representing cells originating from the endoderm, mesoderm, and ectoderm, respectively, thus confirming pluripotent tri-lineage differentiation potential. **B.** KaryoStat+ analysis (copy number assay based digital karyotyping) of C53 and C70 iPSCs confirmed that both lines were derived from male tissue, and did not indicate any chromosomal aberrations, with a detection limit of 1 Mb. A whole genome view of copy number is included and all somatic and sex chromosomes are represented; the pink, green and yellow bands indicate the raw signal for each chromosome, and the blue signal represents the normalized probe signal. A value of 2 in the y-axis represents a normal copy number state, a value of 3 represents a chromosomal gain, and a value of 1 represents a chromosomal loss. **C**. Astrocyte progenitor cells derived from C53 and C70 iPSCs were positive for the astrocyte progenitor marker vimentin by immunofluorescence. **D,E**. HSPB1 was detected in iPSC-derived astrocyte progenitors and mature astrocytes by Western blotting. **F.** Immunolabelling with antibodies targeting GFAP and HSPB1 showed expression of HSPB1 in human iPSC-derived mature astrocytes.

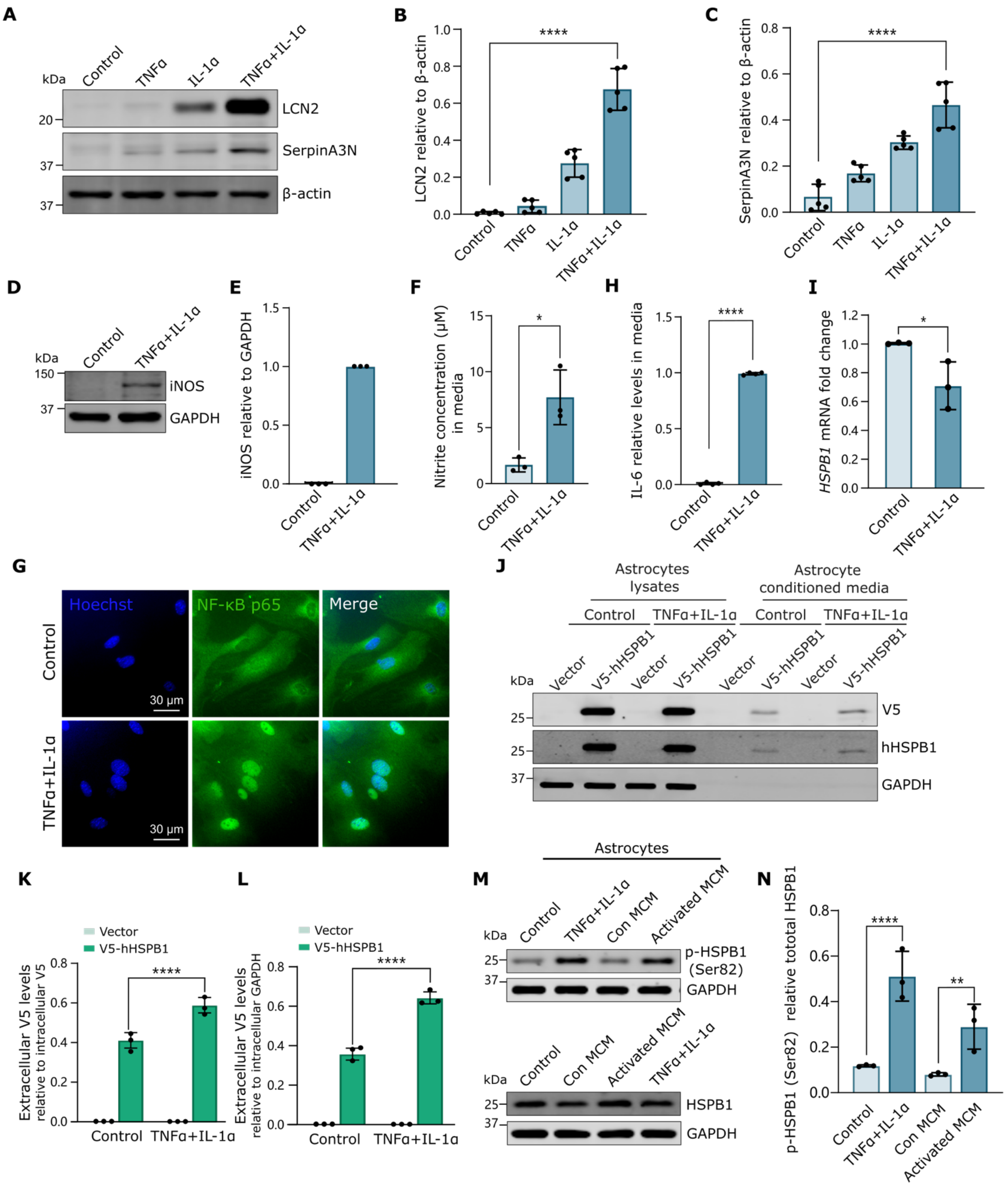
A
Control
TNFα
IL-1α
TNFα+IL-1α
kDa
LCN2
SerpinA3N
β-actin
B
LCN2 relative to β-actin
C
SerpinA3N relative to β-actin
D
iNOS
GAPDH
E
iNOS relative to GAPDH
F
Nitrite concentration (μM) in media
H
IL-6 relative levels in media
I
HSPB1 mRNA fold change
G
Hoechst
NF-κB p65
Merge
30 μm
J
Astrocytes lysates
Astrocyte conditioned media
Vector
V5-hHSPB1
V5
hHSPB1
K
Extracellular V5 levels relative to intracellular V5
L
Extracellular V5 levels relative to intracellular GAPDH
M
Astrocytes
Con MCM
Activated MCM
p-HSPB1 (Ser82)
HSPB1
N
p-HSPB1 (Ser82) relative tototal HSPB1

Figure S5. Modelling inflammatory astrocyte reactivity by treating primary astrocytes with TNFα+IL-1α cytokines. **A-H.** Primary mouse astrocytes were treated with either TNFα (30 ng/ml), IL-1α (3 ng/ml) or TNFα+IL-1α for 24h, as indicated, and intracellular LCN2 (**A,B**), SerpinA3N (**A,C**) or iNOS (**D,E**) were detected by Western blotting and quantified relative to β-actin as a loading control; the concentration of nitrites in the media, as an indirect measure of nitric oxide (NO) levels, was measured using a Griess colorimetric assay (**F**); nuclear translocation of p65, as a readout of NF-kB activation, was observed by immunofluorescence (**G**); and levels of IL-6 in the media were determined by ELISA (**H**). **I**. HSPB1 mRNA levels were quantified relative to GAPDH in control or TNFα+IL-1α conditions. **J-L**. Astrocytes were transfected with either an empty vector or V5-hHSPB1 for 24h followed by treatment with TNFα+IL-1α for further 24h, and V5 or human HSPB1 (hHSPB1) were detected by Western blotting in either astrocyte lysates or concentrated conditioned media. The levels of V5 in media were quantified relative to intracellular V5 (**K**) or intracellular GAPDH (**L**). **M,N**. Levels of phospho-HSPB1 (S82) were detected in astrocytes treated with either TNFα+IL-1α or microglia conditioned media and quantified relative to total HSPB1 in the same sample (**N**). Data are shown as mean ± SD and were analysed by one-way ANOVA with Tukey's multiple comparisons (**B,C,N**), unpaired Student's t-test (**F,H**), one sample t-test (I) or two-way ANOVA with Tukey's multiple comparisons (**K,L**) in a minimum of 3 biological replicates, * p<0.05, **** p<0.0001, ns non significant.

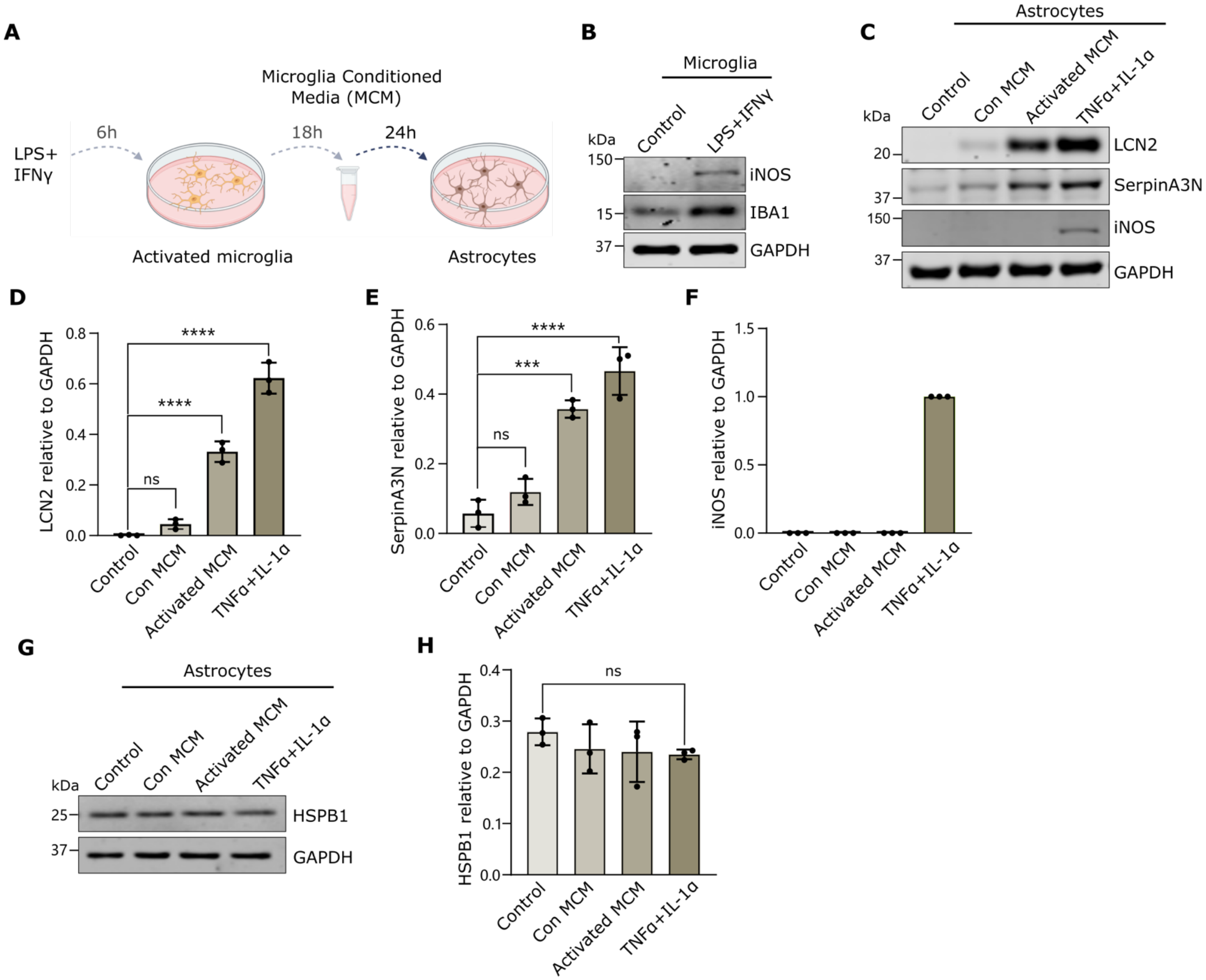

Figure S6. Comparison of the astrocyte response to media from activated microglia and to proinflammatory cytokines. A. Primary microglia from wild type mice were treated with LPS and IFN-γ or kept untreated as control for 6h. Media was then replaced with fresh media and collected after 18h. Microglia conditioned media (MCM) from either control or activated microglia was used to treat primary mouse astrocytes for 24h. Created with BioRender.com. **B**. Microglia cell lysates were subjected to Western blot for iNOS and IBA1 to confirm microglia activation in response to LPS and IFNγ. **C**. LCN2, SerpinA3N and iNOS were detected in astrocyte lysates treated with media from microglia in control conditions (con MCM) or after activation with LPS+IFNγ (activated

MCM), or untreated (control) and compared to TNFα+IL-1α treated astrocytes. The levels of LCN2 (**D**), SerpinA3N (**E**) and iNOS (**F**) were quantified relative to GAPDH. **G,H**. HSPB1 protein levels were detected in astrocyte lysates in the above conditions (**G**) and quantified relative to GAPDH (**H**). Data are shown as mean ± SD (N=3). One-way ANOVA with Tukey's multiple comparisons test (**D,E,H**), *** $p<0.001$, **** $p<0.0001$, ns non significant.

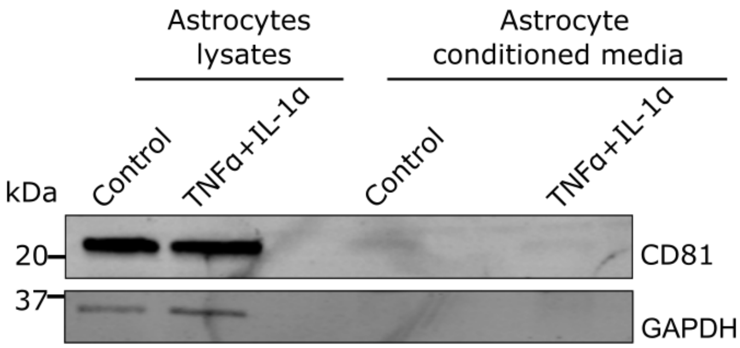

Figure S7. Levels of CD81 do not change in conditioned media of astrocytes treated with TNFα+IL-1α. Levels of CD81 were detected in either astrocyte lysates or concentrated conditioned media of astrocytes treated with control or TNFα+IL-1α for 24h.

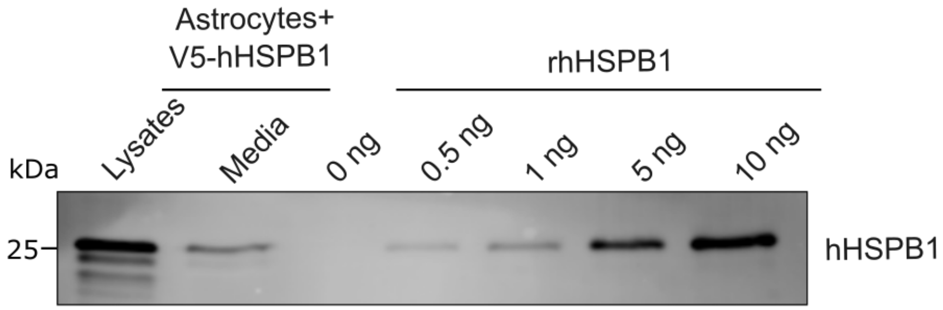

Figure S8. Estimation of HSPB1 concentration in astrocyte media. Concentrated media (equivalent to 0.2 mL of media) from mouse astrocytes expressing V5-hHSPB1 and cultured for 24h were compared to known amounts of recombinant human HSPB1 (rhHSPB1). Approximately, 5 ng/mL of V5-hHSPB1 is present in the media of astrocytes expressing V5-hHSPB1.

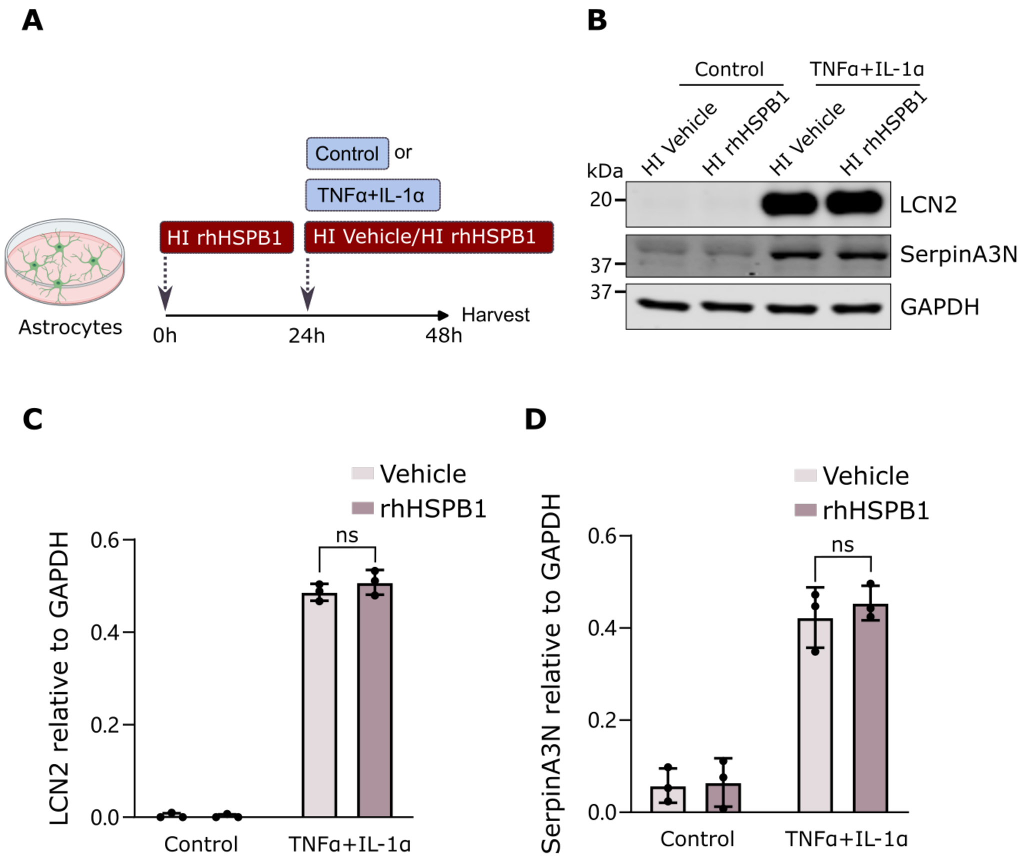

Figure S9. Heat inactivated recombinant human HSPB1 does not attenuate the astrocyte inflammatory response. A. Primary mouse astrocytes were pretreated with heat inactivated (HI) rhHSPB1 for 24h prior to treatment with either HI 0.1% BSA (vehicle) or 50 ng/mL HI rhHSPB1 and either TNFα+IL-1α or control for further 24h. **B-D**. LCN2, SerpinA3N and iNOS were detected by Western blotting of astrocyte lysates and levels quantified relative to GADPH as a loading control. Data are shown as mean ± SD (N=3). Two-way ANOVA with Tukey's multiple comparisons test, ns non significant.

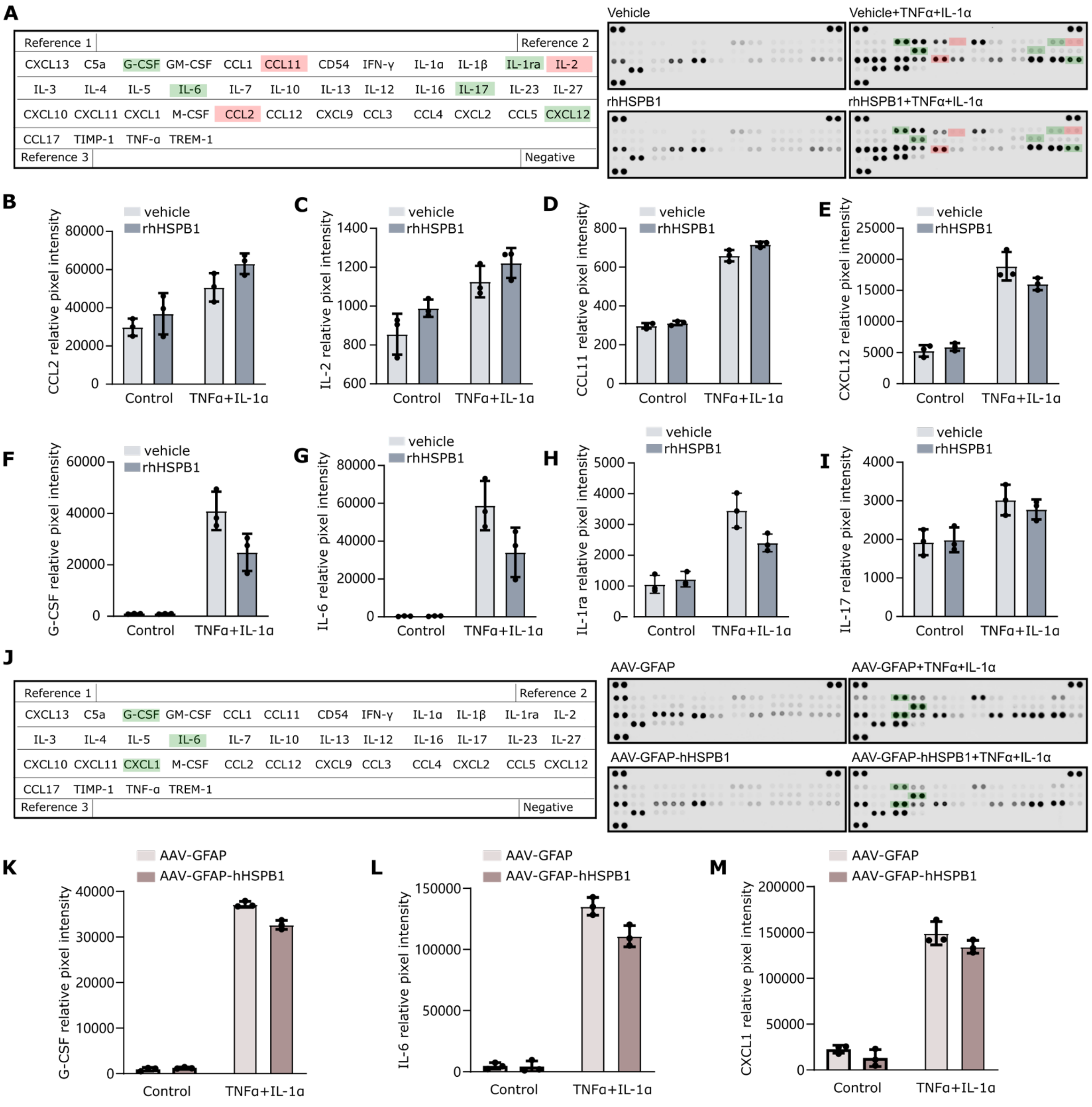

Figure S10. Cytokine arrays to evaluate changes in primary mouse astrocytes and organotypic brain slice cultures in response to HSPB1. **A-I.** Cytokine arrays for astrocyte conditioned media. **A**. Panel of membrane-based antibody array where each cytokine is spotted

in duplicate. Representative membranes incubated with either control/vehicle, control/rhHSPB1, TNFα+IL-1α /vehicle or TNFα+IL-1α /rhHSPB1 are shown. Cytokines that show a significant decrease (green) or increase (red) in TNFα+IL-1α/rhHSPB1 vs TNFα+IL-1α/vehicle are highlighted. The average of pixel intensities of 3 biological replicates is shown for those cytokines that show significant changes in z-score values: CCL2 (**B**), IL-2 (**C**), CCL11 (**D**), CXCL12 (**E**), G-CSF (**F**), IL-6 (**G**), IL-1ra (**H**), IL-17 (**I**). **J-M,** Cytokine arrays for conditioned media from organotypic brain slices. **J**. Panel of membrane-based antibody array where each cytokine is spotted in duplicate. Representative membranes incubated with either control/AAV-GFAP, control/AAV-GFAP-hHSPB1, TNFα+IL-1α /AAV-GFAP or TNFα+IL-1α /AAV-GFAP-hHSPB1 are shown, and cytokines with a significant decrease (green) in TNFα+IL-1α/AAV-GFAP-hHSPB1 vs TNFα+IL-1α /AAV-GFAP are highlighted. The average of pixel intensities of 3 biological replicates is shown for those cytokines that show significant changes in z-score values: G-CSF (**K**), IL-6 (**L**), CXCL1 (**M**). Data are shown as mean ± SD (N=3).

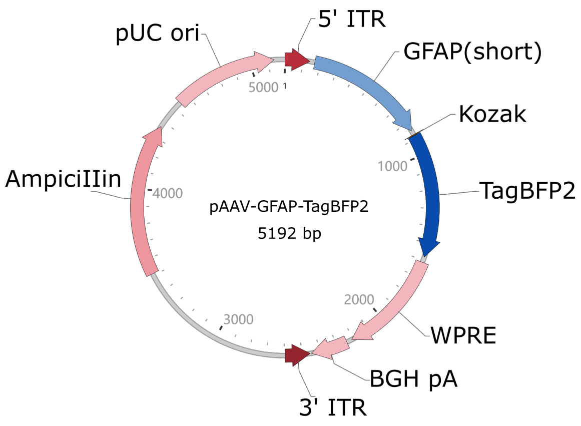

pAAV-GFAP-TagBFP2

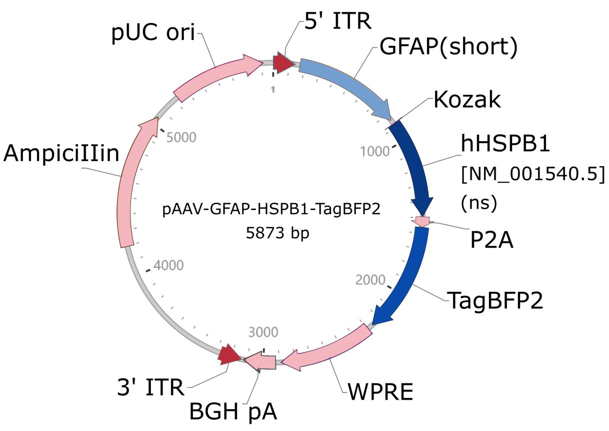

pAAV-GFAP-HSPB1-TagBFP2

Figure S11. Vectors used to generate AAVs to express human HSPB1 under a short form of the GFAP promoter. A blue fluorescent protein (TagBFP2) tag is also expressed with a P2A self-cleaving linker to result in separated translation from hHSPB1.

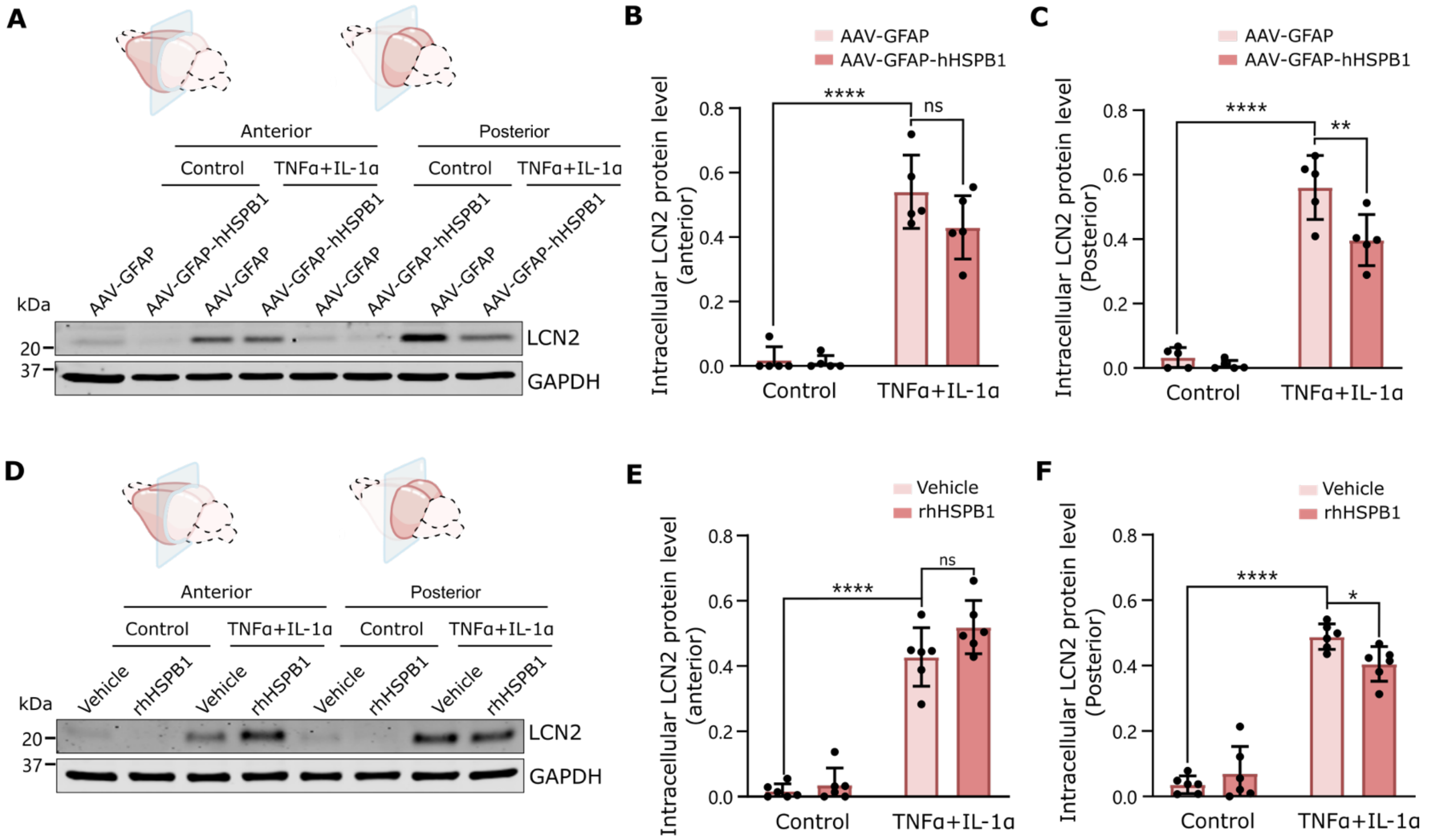

Figure S12. Brain regional differences in the response to secreted HSPB1. A-C. Intracellular levels of LCN2 were detected in organotypic brain slices transduced with AAV-GFAP or AAV-GFAP-hHSPB1 and treated with TNFα+IL-1α or control. The levels of LCN2 relative to GAPDH were quantified in slices separated from the anterior (**B**) or the posterior (**C**) postnatal mouse brain. **D-E**. Intracellular levels of LCN2 were detected in organotypic brain slices pretreated with 50 ng/mL rhHSPB1 followed by treatment with rhHSPB1 or vehicle (0.1% BSA) and TNFα+IL-1α or control, and the levels of LCN2 relative to GAPDH were quantified in slices separated from the anterior (**E**) or the posterior (**F**) postnatal mouse brain. Data are shown as mean ± SD of N=5 (**B,C**) or N=6 (**E,F**) Two-way ANOVA with Tukey's multiple comparisons test, * p<0.05, ** p<0.01, **** p<0.0001, ns non significant.

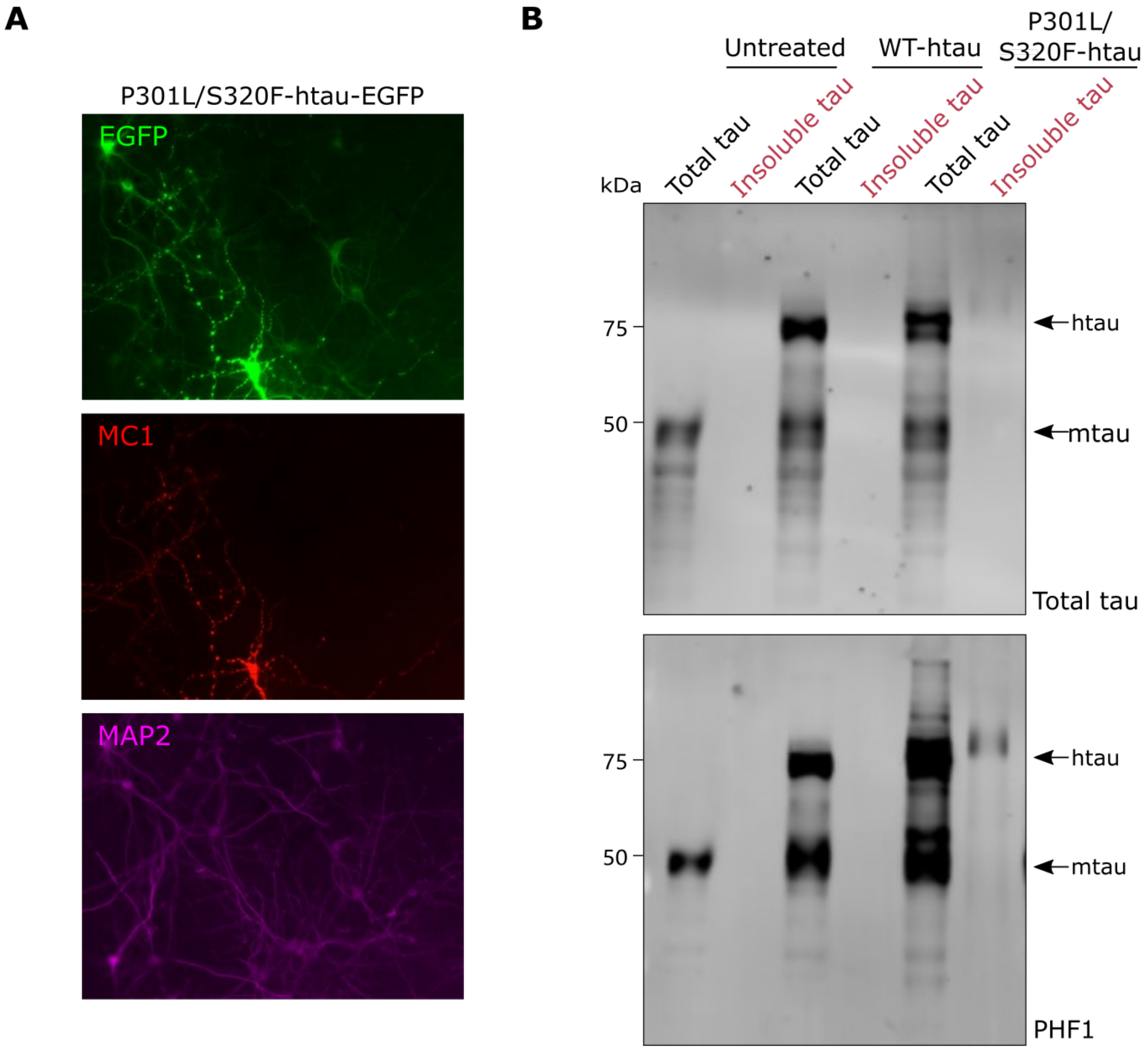

Figure S13. Characterization of P301L/S320F-htau-EGFP inclusions. A. Primary mouse neurons were transduced with AAVs to express mutant P301L/S320F-htau-EGFP at 6 DIV and fixed at 12 DIV, followed by immunofluorescence to confirm that these inclusions were positive for the MC1 antibody that detects tau in an abnormal and disease-associated conformation. **B**. Organotypic brain slices were left untreated or transduced with AAVs to express WT-htau-EGFP or mutant P301L/S320F-htau-EGFP. After 28 DIV, slices were harvested and subjected to sarkosyl extraction to isolate the low-speed supernatant (total tau) or the sarkosyl insoluble pellet (insoluble tau). Fractions were immunoblotted for total tau or Ser396/Ser404 phosphorylated tau (PHF-1).

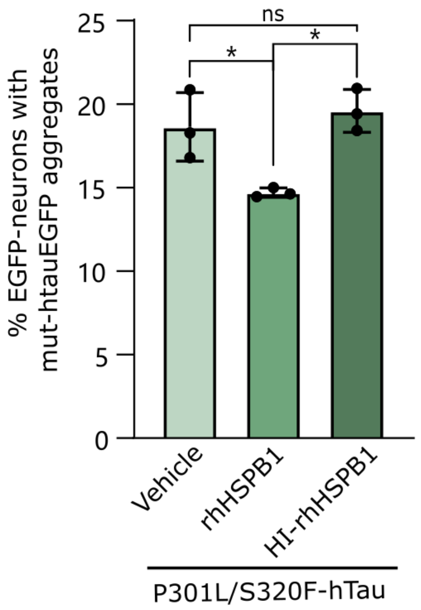

Figure S14. Heat inactivated rhHSPB1 cannot reduce human mutant tau inclusions in primary mouse neurons. Neurons were transduced with AAVs to express mutant P301L/S320F-htau-EGFP at 6 DIV and treated with either vehicle (0.1% BSA), 50 ng/mL rhHSPB1 or 50 ng/mL heat inactivated (HI)-rhHSPB1 every 2 days and fixed at 12 DIV. Graph shows the percentage of EGFP-positive neurons that show mutant htau inclusions. N=3 with >1,500 cell counted per condition in each biological replicate. One-way ANOVA with Tukey's multiple comparisons test * $p<0.05$.

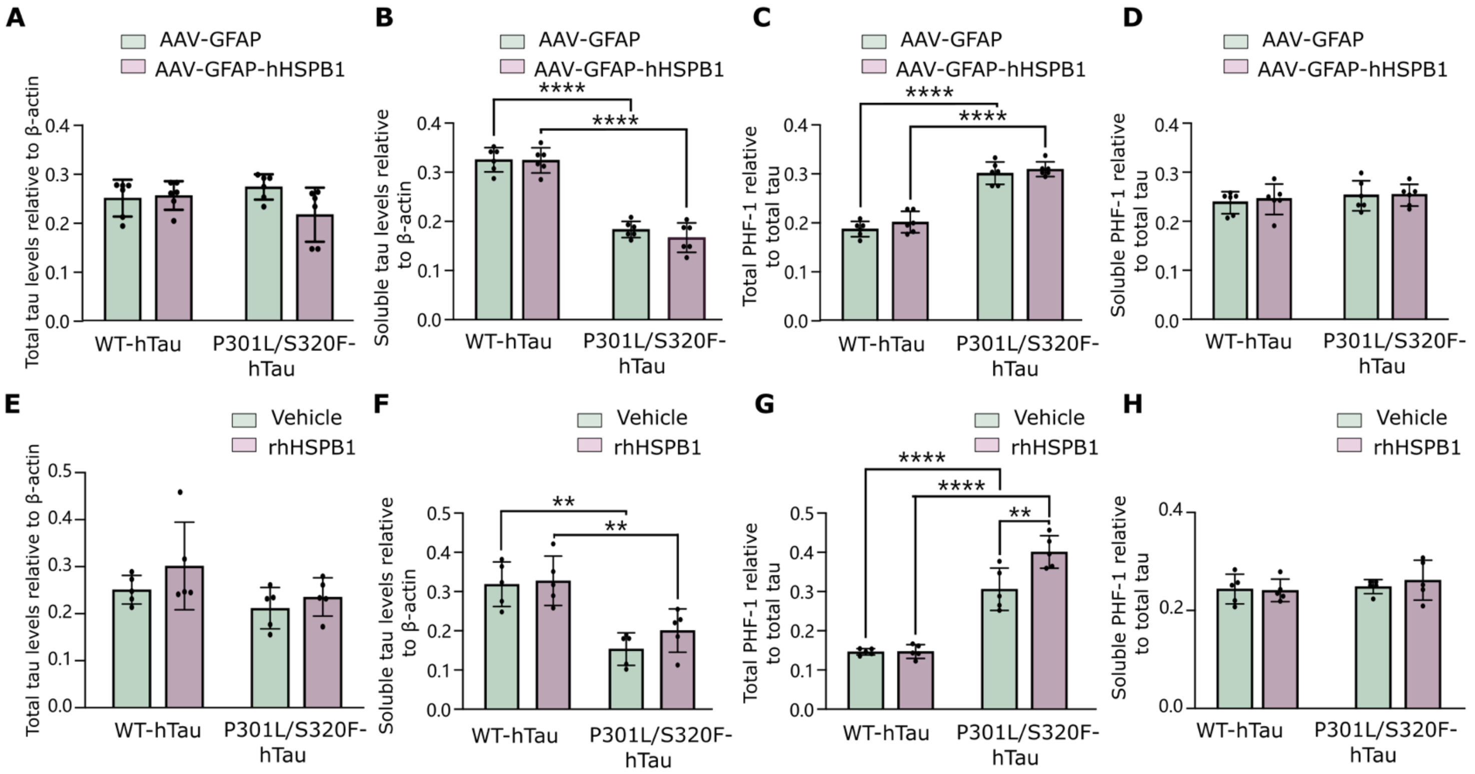

Figure S15. Quantification of tau and PHF-1 in total and soluble fractions of organotypic brain slice cultures after sarkosyl extraction (Quantification of Figure 8). **A-D.** Quantification of tau (**A,B**) or Ser396/Ser404 (PHF1) tau (**C,D**) in total tau fraction (**A,C**) or sarkosyl-soluble fraction (**B,D**) from Fig. 8c. **E-H.** Quantification of tau (**E,F**) or Ser396/Ser404 (PHF1) tau (**G,H**) in total tau fraction (**E,G**) or sarkosyl-soluble fraction (**F,H**) from Fig. 8f. Data are shown as mean ± SD (N=6 (**A-D**), N=5 (**E-H**)). Two-way ANOVA with Tukey multiple comparison test, ** $p<0.01$, **** $p<0.0001$.

Braak stage	SEX	AGE	PMD (h)	Pathology/Diagnosis
**Braak VI**	M	72	41	Alzheimer's disease (modified Braak/BNE stage VI) with extensive and capillary amyloid angiopathy and significant cerebrovascular pathology.
**Braak VI**	F	79	63	Alzheimer's disease BNE stage VI. Marked amyloid angiopathy.
**Braak VI**	F	83	58	Alzheimer's disease Braak stage VI.
**Braak VI**	F	85	12	Alzheimer's disease, BNE stage VI. Moderate cerebral amyloid angiopathy, non-capillary, type. Mild small vessel disease.
**Braak VI**	F	81	20	Alzheimer's disease (Braak stage VI). Vascular disease. Brainstem haemorrhage.
**Braak V**	M	75	59	Alzheimer's Disease (modified Braak (BNE) stage V, Thal phase 5 with moderate-severe amyloid angiopathy.
**Braak IV**	F	82	20	Alzheimer's disease modified Braak IV with amyloid angiopathy. Lewy body pathology (amygdala only).
**Braak IV**	M	81	51	Alzheimer's disease, BNE stage IV. Brainstem predominant lewy body disease, mild amyloid angiopathy.
**Braak IV**	M	84	86	Alzheimer's disease (modified Braak (BNE) stage IV) with moderate amyloid angiopathy.
**Braak IV**	F	86	55.5	Alzheimer's disease (modified Braak (BNE) stage IV) with mild amyloid angiopathy.
**Control/ Braak II**	F	86	45	Alzheimer type changes (ageing), BNE stage II, control. Mild small vessel disease, mild amyloid angiopathy.
**Control/ Braak II**	F	84	53	Alzheimer changes, Braak II, consistent with patient's age, control.
**Control/ Braak II**	F	84	35	Alzheimer changes Braak II consistent with normal aging, control.
**Control/ Braak I-II**	M	73	49	Early Alzheimer-type changes BNE stage I-II control.
**Control/ Braak 0**	M	77	28.5	Mild to moderate amyloid angiopathy, capillary subtype. BNE braak +, CERAD No AD, low VCING; control.

Table S1. List of human post-mortem samples used in this study. The Braak stage, sex (M=Male; F=Female), age in years, postmortem delay (PMD) in hours and the pathology or diagnosis is indicated. All samples refer to temporal cortex. BNE= Brain Net Europe.
